# Small extracellular vesicles promote invadopodia activity in glioblastoma cells in a therapy-dependent manner

**DOI:** 10.1007/s13402-023-00786-w

**Published:** 2023-04-04

**Authors:** Clarissa A. Whitehead, Haoyun Fang, Huaqi Su, Andrew P. Morokoff, Andrew H. Kaye, Eric Hanssen, Cameron J. Nowell, Katharine J. Drummond, David W. Greening, Laura J. Vella, Theo Mantamadiotis, Stanley S. Stylli

**Affiliations:** 1 Department of Surgery, Royal Melbourne Hospital, The University of Melbourne, Parkville, VIC Australia; 2 Molecular Proteomics, Baker Heart and Diabetes Institute, Melbourne, VIC Australia; 3 Centre for Stem Cell Systems, The University of Melbourne, Parkville, VIC Australia; 4 Department of Biochemistry and Pharmacology, The University of Melbourne, Parkville, VIC 3010 Australia; 5 Department of Surgery, The Royal Melbourne Hospital, The University of Melbourne, Level 5, Clinical Sciences Building, Parkville, VIC 3050 Australia; 6 Department of Neurosurgery, Hadassah Hebrew University Medical Centre, Jerusalem, Israel; 7 Advanced Microscopy Facility, The Bio21 Molecular Science and Biotechnology Institute, The University of Melbourne, Parkville, VIC 3010 Australia; 8 Drug Discovery Biology, Monash Institute of Pharmaceutical Sciences, Monash University, Melbourne, VIC 3052 Australia; 9 Baker Department of Cardiovascular Research, Translation and Implementation, La Trobe University, Melbourne, VIC Australia; 10 Central Clinical School, Monash University, Melbourne, VIC Australia; 11 Baker Department of Cardiometabolic Health, University of Melbourne, Melbourne, VIC Australia; 12 Department of Microbiology and Immunology, The University of Melbourne, Melbourne, VIC Australia

**Keywords:** Glioblastoma, Invadopodia, Extracellular vesicles, Radiotherapy, Temozolomide

## Abstract

**Purpose:**

The therapeutic efficacy of radiotherapy/temozolomide treatment for glioblastoma (GBM) is limited by the augmented invasiveness mediated by invadopodia activity of surviving GBM cells. As yet, however the underlying mechanisms remain poorly understood. Due to their ability to transport oncogenic material between cells, small extracellular vesicles (sEVs) have emerged as key mediators of tumour progression. We hypothesize that the sustained growth and invasion of cancer cells depends on bidirectional sEV-mediated cell–cell communication.

**Methods:**

Invadopodia assays and zymography gels were used to examine the invadopodia activity capacity of GBM cells. Differential ultracentrifugation was utilized to isolate sEVs from conditioned medium and proteomic analyses were conducted on both GBM cell lines and their sEVs to determine the cargo present within the sEVs. In addition, the impact of radiotherapy and temozolomide treatment of GBM cells was studied.

**Results:**

We found that GBM cells form active invadopodia and secrete sEVs containing the matrix metalloproteinase MMP-2. Subsequent proteomic studies revealed the presence of an invadopodia-related protein sEV cargo and that sEVs from highly invadopodia active GBM cells (LN229) increase invadopodia activity in sEV recipient GBM cells. We also found that GBM cells displayed increases in invadopodia activity and sEV secretion post radiation/temozolomide treatment. Together, these data reveal a relationship between invadopodia and sEV composition/secretion/uptake in promoting the invasiveness of GBM cells.

**Conclusions:**

Our data indicate that sEVs secreted by GBM cells can facilitate tumour invasion by promoting invadopodia activity in recipient cells, which may be enhanced by treatment with radio-chemotherapy. The transfer of pro-invasive cargos may yield important insights into the functional capacity of sEVs in invadopodia.

**Supplementary Information:**

The online version contains supplementary material available at 10.1007/s13402-023-00786-w.

## Introduction

Glioblastoma (GBM) is the most common and aggressive primary brain tumour in adults, d is highly infiltrative and uniformly lethal [1]. Despite aggressive therapeutic intervention with surgery, radiotherapy (RT) and chemotherapy with oral temozolomide (TMZ), the prognosis for GBM patients remains dismal due the inevitability of tumour recurrence [2, 3]. Emerging evidence suggests that radio- and chemo-therapeutic stress can reprogram tumour cells, endowing them with the ability to generate more invasive recurrent tumours [4]. While tumour recurrence occurs because of the presence of GBM cells that survive treatment, several studies have indicated that the efficacy of RT/TMZ treatment in GBM is further compromised by the surviving tumour cells that exhibit enhanced invasive capabilities compared to the untreated cells, resulting in tumour recurrence away from the original site of tumour debulking [5–9]. Although inhibition of the enhanced invasiveness of surviving GBM cells may improve patient outcome, the mechanisms utilised by invasive GBM cells following treatment are not well understood.

To facilitate invasion, tumour cells form actin-rich membrane protrusions known as invadopodia, which utilize transmembrane proteases, such as MT1-MMP, and secreted proteases, such as MMP-2, to degrade the surrounding extracellular matrix (ECM) [10]. In addition to remodelling the ECM, this proteolytic activity results in the cleavage of non-matrix targets including latent cytokines or integrins, which may also enhance tumour growth via the activation of pro-invasive signalling pathways. As GBM cells can form matrix degrading invadopodia [11–13], the enhanced invasive phenotype of GBM cells following RT/TMZ treatment may be mediated via invadopodia. Previously, we have shown that invadopodia activity is enhanced in GBM cells that survive RT/TMZ treatment, but the underlying mechanisms remained unclear [12, 14].

Additionally, tumour growth and invasion may be mediated through intercellular paracrine signalling by extracellular vesicles (EVs) [15]. EVs are small membrane-enclosed particles that are secreted to mediate the transfer of DNA, RNA, proteins and lipids between cells [15]. Recent studies have highlighted a crucial role for a major subset of EVs called small EVs (sEVs; 30–200 nm in diameter) in GBM through their ability to transfer oncogenic molecular cargo to modulate the composition and function of target cells [16–19]. But, as yet, the full extent by which GBM-derived sEVs can drive GBM cell invasion or growth in response to therapy has not been fully elucidated.

In this study, we report a paracrine signalling loop whereby GBM cell-derived sEVs carrying invadopodia-associated proteins can functionally induce invadopodia in recipient GBM cells. Using clinically relevant doses of RT and TMZ, we show that RT/TMZ treatment of GBM cells leads to enhanced sEV secretion, augmented invadopodia formation and FITC-gelatin degrading activity, and results in an altered proteomic landscape that supports a pro-invadopodia and invasive phenotype. Our findings may have important implications in understanding oncogenic sEVs in promoting cell invasiveness and invadopodia.

## Materials and methods

### Cell lines and culture conditions

Human GBM cell lines U87MG and LN229 were obtained from the ATCC Biological Material Repository. Primary GBM cell lines MU4 and MU41 were generated from GBM patient biopsy specimens acquired during surgery performed at The Royal Melbourne Hospital (Human Research Ethics Committee Approval Number: HREC 2009.016 – informed consent was provided by the patients). The cells were cultured in DMEM supplemented with 10% heat inactivated FBS, penicillin (100 U/ml), and streptomycin (10 μg/ml). All cell lines were mycoplasma free and were maintained in a humidified atmosphere of 5% CO_2_ at 37 °C and utilized within the first 20 cell passages.

### Antibodies and reagents

Monoclonal anti-Alix (ab117600) and anti-Calnexin (ab22595) antibodies were purchased from Abcam, whilst polyclonal anti-β-tubulin (#2146) was purchased from Cell Signalling Technologies. Secondary antibodies (rabbit- and mouse- anti-goat IgG HRP conjugate #170–6515 and #170–6516) were purchased from Bio-Rad. Rhodamine-conjugated phalloidin (PHDR1) was purchased from Cytoskeleton and DAPI (D9542) was purchased from Sigma-Aldrich. DMEM, OptiMEM and fetal bovine serum (FBS) were purchased from Thermofisher Scientific. Vinorelbine tartrate (#S4269) was purchased from Selleckchem.

### Gelatinase zymography

GBM cells were seeded at 2 × 10^5^ cells per well in 6-well plates and incubated in serum-free OptiMEM for 24 h in a humidified atmosphere of 5% CO_2_ at 37 °C prior to harvesting conditioned media. Cells were lysed (50 mM Tris (pH 7.4), 150 mM NaCl, 1% Triton X-100, 50 mM NaF, 2 mM MgCl_2_, 1 mM Na_3_VO_4_ and protease inhibitor cocktail (Roche)) and cleared by centrifuging at 13,000xg at 4 °C, after which protein concentrations were determined using a BCA protein assay (Thermofisher Scientific). Conditioned serum-free OptiMEM medium aliquots (100 μl) were centrifuged at 1000xg (4 °C) for 10 min to remove cell debris and separated by gel electrophoresis (Novex 10% Zymogram Plus) (Thermofisher Scientific). Sample loading of the conditioned media was normalised relative to the protein concentration of the corresponding cell lysates. Following electrophoresis, gels were incubated in Novex Zymogram renaturing and developing buffers as per manufacturer’s instructions (Thermofisher Scientific), before a final overnight incubation in developing buffer at 37 °C. Gels were then stained with SimplyBlue® (Thermofisher Scientific) for the detection of clear gelatinolytic bands. Band intensities were quantitated using ImageJ (Version 1.53a).

### Invadopodia-mediated FITC-gelatin degradation assay

Autoclaved coverslips were coated in fluorescein isothiocyanate (FITC)-conjugated gelatin, as described before [20], and incubated for 2 h at 37 °C in serum-free DMEM. Cells were seeded onto the coated coverslips in DMEM supplemented with 5% FBS and incubated overnight at 37 °C (5% CO_2_). Cells were then washed and fixed in 4% paraformaldehyde in PBS, permeabilized (0.2% Triton-X-100 in PBS) and stained with PHDR1 (1:75) to visualize actin puncta identifying the invadopodium core, followed by nuclear staining using DAPI (5 μg/ml). Coverslips were mounted on microscope slides with Vectashield Antifade mounting medium and images were acquired with a Nikon A1 + Confocal microscope system (405, 488 and 532 nm lasers) utilizing a Plan Apo VC 60 × Oil DIC N2 immersion objective at a resolution of 1024 × 1024 pixels^2^ and a 1 × zoom factor. The area of FITC-gelatin degradation was normalized relative to the number of cells (DAPI positive nuclei) present within the image using ImageJ (Version 1.53a). A customised script for ImageJ (Version 1.53a) was used to determine the total number actin puncta per image and the puncta which overlapped with areas of degraded FITC-gelatin within cells to quantify both invadopodia formation and activity.

### Differential ultracentrifugation isolation of sEVs

sEVs were isolated as described before [21]. Cells were grown in culture media until ~ 60% confluency (1 × 10^7^ cells per 15 cm dish), washed with sterile PBS and incubated for 24 h in serum-free OptiMEM. The conditioned media were then collected and clarified to remove detached cells (300xg, 10 min) followed by the removal of cell debris (2000xg, 15 min), using a benchtop centrifuge at 4 °C. The supernatants were then transferred to ultra-clear SW40Ti tubes (Beckman Coulter) and ultracentrifuged at 10,000xg for 30 min at 4 °C using a SW40Ti rotor (Beckman Coulter) to pellet large EVs/shed microvesicles. The supernatant was then ultracentrifuged at 100,000xg for 1 h at 4 °C to pellet sEVs, which were subsequently washed in sterile filtered PBS and subjected to an additional ultracentrifugation step at 100,000xg for 1 h at 4 °C (SW40Ti rotor, Beckman Coulter). The resulting sEV pellets were resuspended in 50 μl of filtered PBS, aliquoted and characterized according to the guidelines by the International Society for Extracellular Vesicles [22]. sEV aliquots were either used fresh or stored at -80 °C. For experiments comparing cell and sEV lysates, sEVs were lysed in an equal volume of lysis buffer, whilst 2 × 10^6^ cells were harvested at the time of sEV isolation and lysed in 100 µl lysis buffer (50 mM Tris (pH 7.4), 150 mM NaCl, 1% Triton X-100, 50 mM NaF, 2 mM MgCl_2_, 1 mM Na_3_VO_4_ and protease inhibitor cocktail (Roche). Protein quantitation was then performed using a Pierce ™ BCA Protein Assay Kit according to the manufacturer’s protocol.

### Characterization of sEVs

#### Single nanoparticle analysis

The size distribution and particle concentration of sEVs were determined using nanoparticle tracking analysis (NTA) (NanoSight NS300, Malvern), as described before [23]. The particle concentration was normalised against the corresponding cell count for each sample.

#### Cryo-electron microscopy

Size distribution and morphology assessment of sEVs using cryo-electron microscopy (CryoEM) was performed as described before [24]. Briefly, a 3 μl aliquot of the 50 µl sEV preparation from each GBM cell line (prepared fresh on the day of analysis) was applied to holey carbon grids (ProSciTech), and excess liquid was removed before the grids were plunge-frozen in liquid ethane. The grids were then mounted in a Gatan cryoholder (Gatan) which was pre-cooled in liquid nitrogen. Images were acquired at 200 kV using a Tecnai F30 (FEI) Transmission Electron Microscope. N = 2.

#### Western blot analysis

20 μg of GBM cell or sEV lysates were resolved by SDS-PAGE using NuPage 4–12% Bis–Tris precast gels (Invitrogen) and transferred onto nitrocellulose membranes (GE Healthcare). The membranes were blocked with 3% bovine serum albumin in 1% TBST for 1 h prior to an overnight incubation at 4 °C with primary antibodies (including EV marker ALIX/non-EV marker calnexin, diluted at 1:1000). The membranes were subsequently incubated with the appropriate secondary antibodies (diluted at 1:10,000) and developed using enhanced chemiluminescence reagent (GE Healthcare).

### Sample preparation and proteomic profiling of the GBM cell and sEV proteome

Quantitative data-dependent acquisition mass spectrometry of the LN229, MU4 and MU41 GBM cell lines and sEVs was performed as previously described [24], n = 2. Samples were solubilised in sodium dodecyl sulphate (SDS) 1% (v/v), 50 mM triethylammonium bicarbonate (TEAB), pH 8.0, centrifuged at 16,000xg for 20 min at 4 °C and quantified by microBCA (Life Technologies). For mass spectrometry-based proteomics, samples (5 µg) were normalized and reduced with 10 mM dithiothreitol (DTT) for 45 min at 50 °C followed by alkylation with 20 mM iodoacetamide for 30 min at 25 °C in the dark. The reaction was quenched to a final concentration of 20 mM DTT. Lysates were precipitated with six volumes of acetone overnight at -20 °C. Protein pellets were centrifuged at 16,000xg, 10 min at 4 °C and resuspended in 50 mM TEAB, pH 8.0. Samples were digested with trypsin (Promega, V5111) at a 1:50 enzyme-to-substrate ratio for 16 h at 37 °C. The peptide mixture was acidified to a final concentration of 2% formic acid and centrifuged at 16,000xg for 5 min, frozen at -20 °C for 30 min, and dried by vacuum centrifugation. Peptides were resuspended in 0.07% trifluoroacetic acid (TFA) TFA in MS-grade water. The peptide solutions were acidified to a final concentration of 1% formic acid (FA) and 0.1% triflouroacetic acid (TFA) and desalted with a µC18 Sep-Pak column/plate (Waters). Each Sep-Pak column was activated with 100 µl methanol, washed with 30 µl 80% acetonitrile, and equilibrated with 3 × 30 µl 0.1% TFA. Samples were loaded and each column was washed with 2 × 20 µl 0.1% TFA. Elution was performed with two rounds of 20 μl 50% acetonitrile. Samples were lyophilised (SpeedVac; Savant, ThermoFisher Scientific), acidified with 0.1% FA, 2% ACN, and quantified by Fluorometric Peptide Assay (Thermofisher Scientific, 23,290) as per the manufacturer’s instructions, and normalized to 1 µg per 3 µl.

Peptides were analysed on a Dionex UltiMate NCS-3500RS nanoUHPLC coupled to a Q-Exactive HF-X hybrid quadrupole-Orbitrap mass spectrometer equipped with a nanospray ion source in positive mode [25, 26]. Peptides were loaded (Acclaim PepMap100 C18 3 μm beads with 100 Å pore-size, Thermofisher Scientific) and separated (1.9 µm particle size C18, 0.075 × 150 mm, Nikkyo Technos Co. Ltd) with a gradient of 2–28% acetonitrile containing 0.1% formic acid over 45 min followed by 28–80% from 45–47 min for total runtime of 56 min at 300 nl min-1 at 55 °C (butterfly portfolio heater, Phoenix S&T). An MS1 scan was acquired from 300–1,650 m/z (60,000 resolution, 3 × 10^6^ automatic gain control (AGC), 128 ms injection time) followed by MS/MS data-dependent acquisition (top 30) with collision-induced dissociation and detection in the ion trap (15,000 resolution, 1 × 10^5^ AGC, 25 ms injection time, 28.5% normalized collision energy, 1.3 m/z quadrupole isolation width). Unassigned precursor ions charge states and slightly charged species were rejected and peptide match disabled. Selected sequenced ions were dynamically excluded for 30 s. Data were acquired using Xcalibur software v4.0 (Thermofisher Scientific). A list of samples and RAW data is available in ProteomeXchange Consortium via the PRIDE partner repository; #PXD031077.

### Data processing and bioinformatics pipeline

Peptide identification and quantification were performed using MaxQuant (v1.6.6.0) with its built-in search engine Andromeda [27]. Tandem mass spectra were searched against the *Homo sapiens* (human) reference proteome (74,788 entries, downloaded 11–2019) supplemented with common contaminants. Search parameters included carbamidomethylated cysteine as fixed modification and oxidation of methionine and N-terminal protein acetylation as variable modifications. Data were processed using trypsin/P as the proteolytic enzyme with up to two missed cleavage sites allowed. Precursor mass tolerance was 20 ppm; product ions were searched at 0.15 Da tolerances; and minimum peptide length was defined at 6, maximum peptide length at 144, and max delta CN at 0.05, with a 1% false discovery rate on protein and peptide spectrum match (PSM) level employing a target-decoy approach [28]. ‘Match between run algorithm’ was performed and label free quantification (LFQ) algorithm in MaxQuant (maxLFQ; matching time window 0.7, ion mobility window 0.05, alignment time 20 min) to obtain quantification intensity values. Perseus was used to quantify proteins whose expression was identified in at least 50% in at least one group [29]. LFQ intensities were log2 transformed after removing contaminants and reverse identifications. Proteins were subjected to a two-tail student’s t-test with *p* value adjusted at 5% permutation-based [30].

### Functional enrichment and gene ontology analysis

Functional enrichment analysis of over-represented networks and gene ontology (GO) terms were performed using FunRich (v 3.1.3) [31]. *P* values were calculated by a two-sided hypergeometric test, in addition to Bonferroni and Benjamini–Hochberg (also known as false discovery rate ‘FDR’) corrections for multiple testing.

### Correlation of GBM cell and sEV proteomes with GBM patient survival

GBM patient tumour gene expression and associated survival data from The Cancer Genome Atlas (TCGA) were examined using the Glioblastoma Bio Discovery Portal (http://gbm-biodp.nci.nih.gov) [32], which integrates GBM patient survival data with mRNA expression datasets (Affymetrix HGU133A, Agilent G4502A, HuEx-1_0-st-v2, 3-Platform Aggregates). A Cox proportional hazards model was constructed based on a prognostic index generated from the combined expression levels of the interrogated genes and data were stratified according to the lowest quartile (QT) (blue – lowest expression) versus the highest quartile (QT) (red – highest expression). *P* values below *p* = 0.01 are listed as ‘p-val = 0’.

### Ivy glioblastoma anatomic transcriptional atlas analysis of GBM cell lines and sEV proteomes

**Fig. 1:**
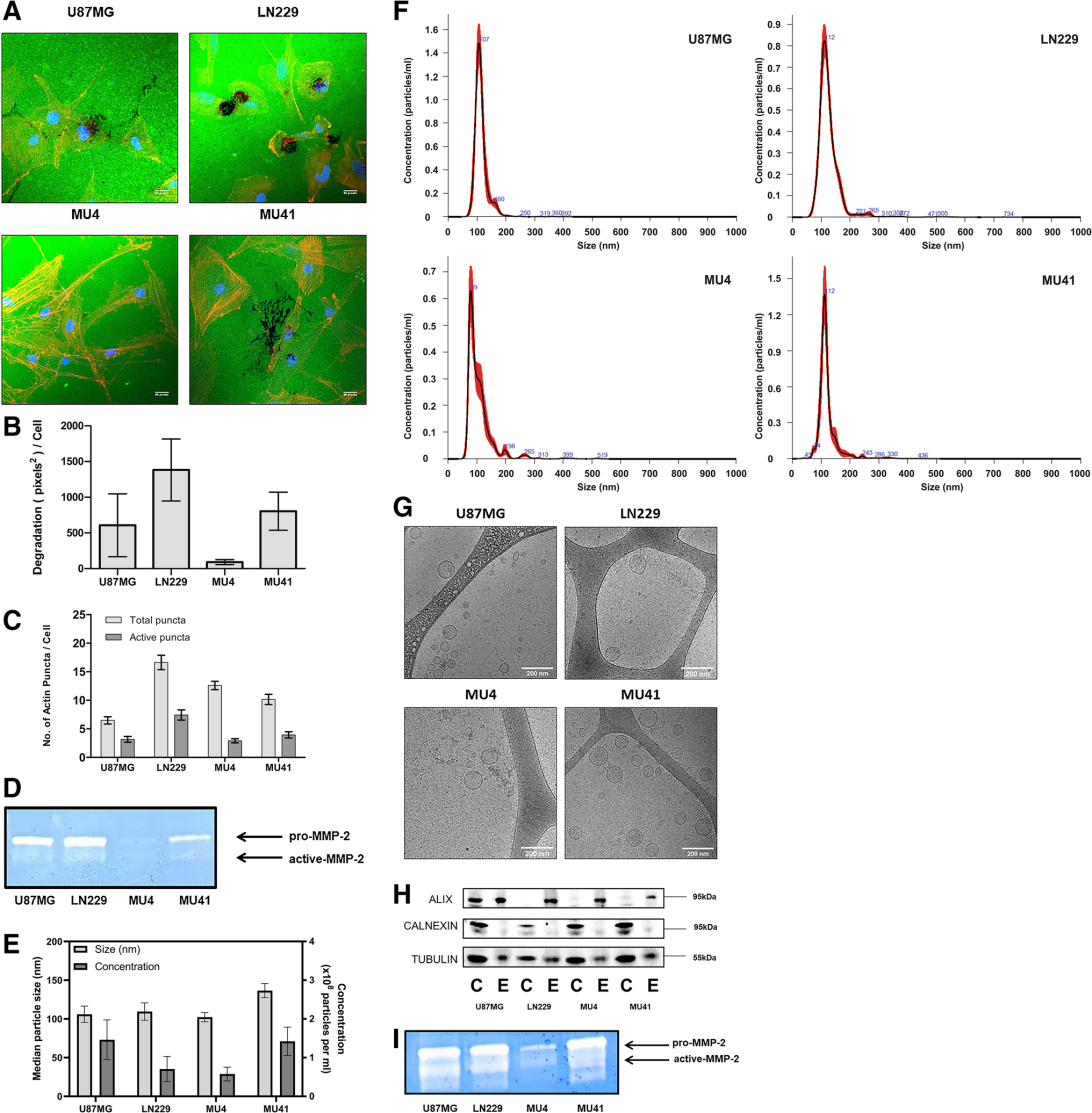
Glioblastoma cells form FITC-gelatin degrading invadopodia and secrete small extracellular vesicles. **(A)** U87MG, LN229, MU4 and MU41 GBM cells were cultured on FITC-gelatin (green) coated coverslips for 24 h, fixed and stained for phalloidin (orange) and DAPI (blue). Scale bar, 33 μm. **(B)** Quantification of FITC-gelatin degradation and **(C)** invadopodia (as rhodamine phalloidin stained actin puncta) normalized to the number of cells present in each image as determined by the number of nuclei (DAPI). **(D)** MMP-2 is the only detectable secreted protease from GBM cells using gelatin-based zymography (representative image from n = 3 experiments). **(E)** Nanoparticle Tracking Analysis (NTA) of sEVs isolated from GBM cells indicating the median particle size and average concentration of sEVs normalized to 1 × 10^5^ donor cells. (**F**) NTA profiles of conditioned medium from GBM cells. The vesicles isolated from the medium are predominantly sEVs (< 200 nm) with minimal presence of larger sized vesicles. **(G)** Cryogenic electron microscopy images confirming the morphology of sEVs and their size range observed with NTA. Scale bar, 200 nm, n = 2. **(H)** Western blot analysis of cell (C) and sEV (E) lysates indicating an enrichment of the EV marker ALIX and a low expression of the ER marker Calnexin in sEVs relative to each donor cell. **(I)** Gelatin-based zymographic analysis indicating the presence of MMP-2 in sEVs of all cell models. Loading volumes were normalized to particle count as determined by NTA (representative image from n = 3 experiments)

**Fig. 2:**
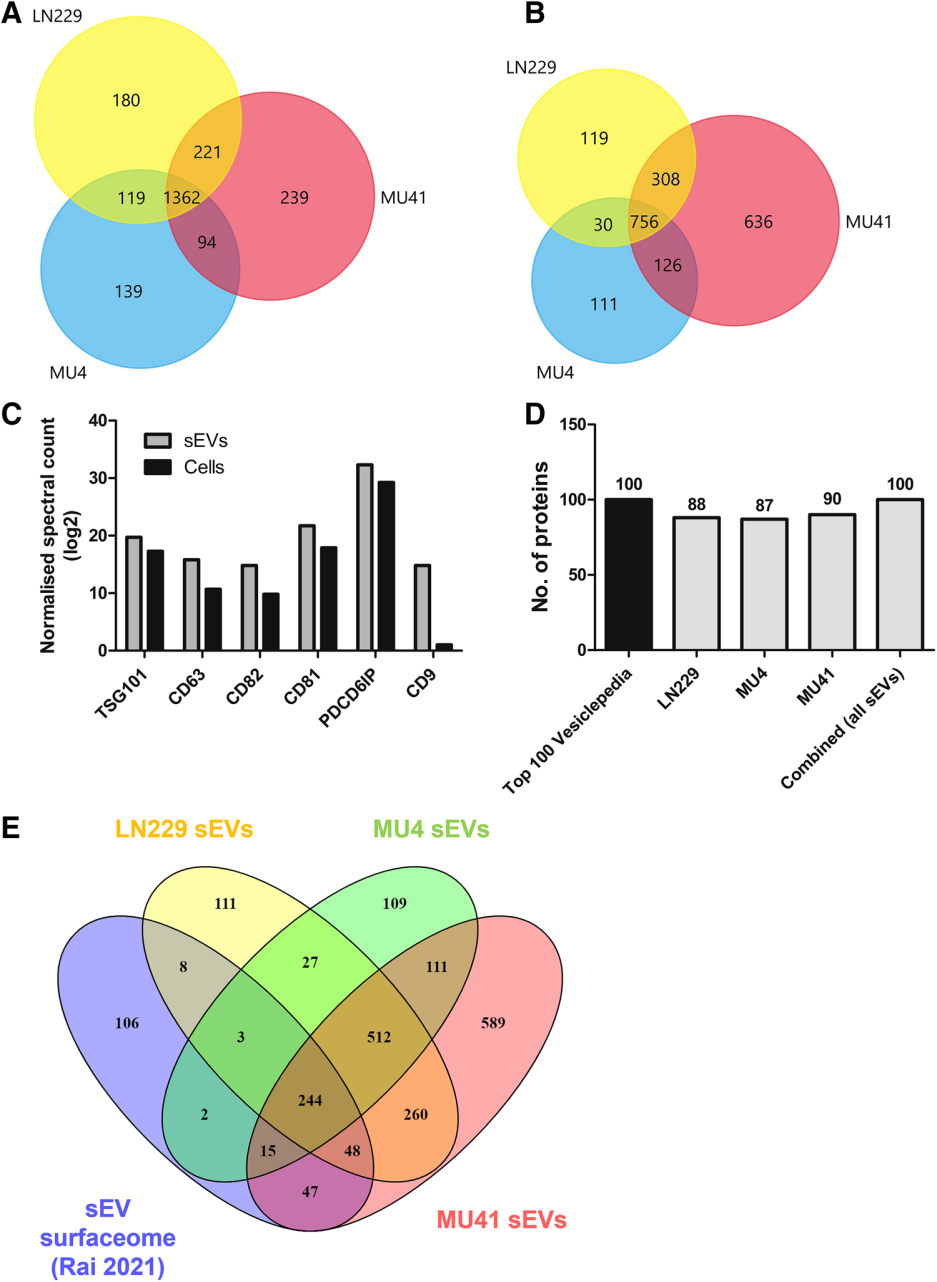
Proteome profiling of GBM cell lines and sEVs. **(A)** Venn diagram of total proteins detected in the GBM cell line (LN229, MU4 and MU41) proteomes. 1362 proteins were common to all three cell lines. **(B)** Venn diagram of the total proteins detected in the GBM cell line-derived sEVs. 756 proteins were common to the sEVs harvested from the three cell lines. **(C)** Elevated expression of exosomal markers CD81, CD82, CD63, CD9, PDCD6IP (ALIX) and TSG101 in sEVs compared to donor GBM cells. **(D)** Number of proteins detected in sEVs from GBM cells or combined cell-derived sEVs from all models that are present in the Top 100 common EV proteins in Vesiclepedia. **(E)** Venn diagram of the sEV ‘surfaceome’ upon comparison of sEV proteomes with the EV surfaceome identified by Rai et al. [43]. 244 surface proteins were common to sEVs from all three cell lines

**Fig. 3:**
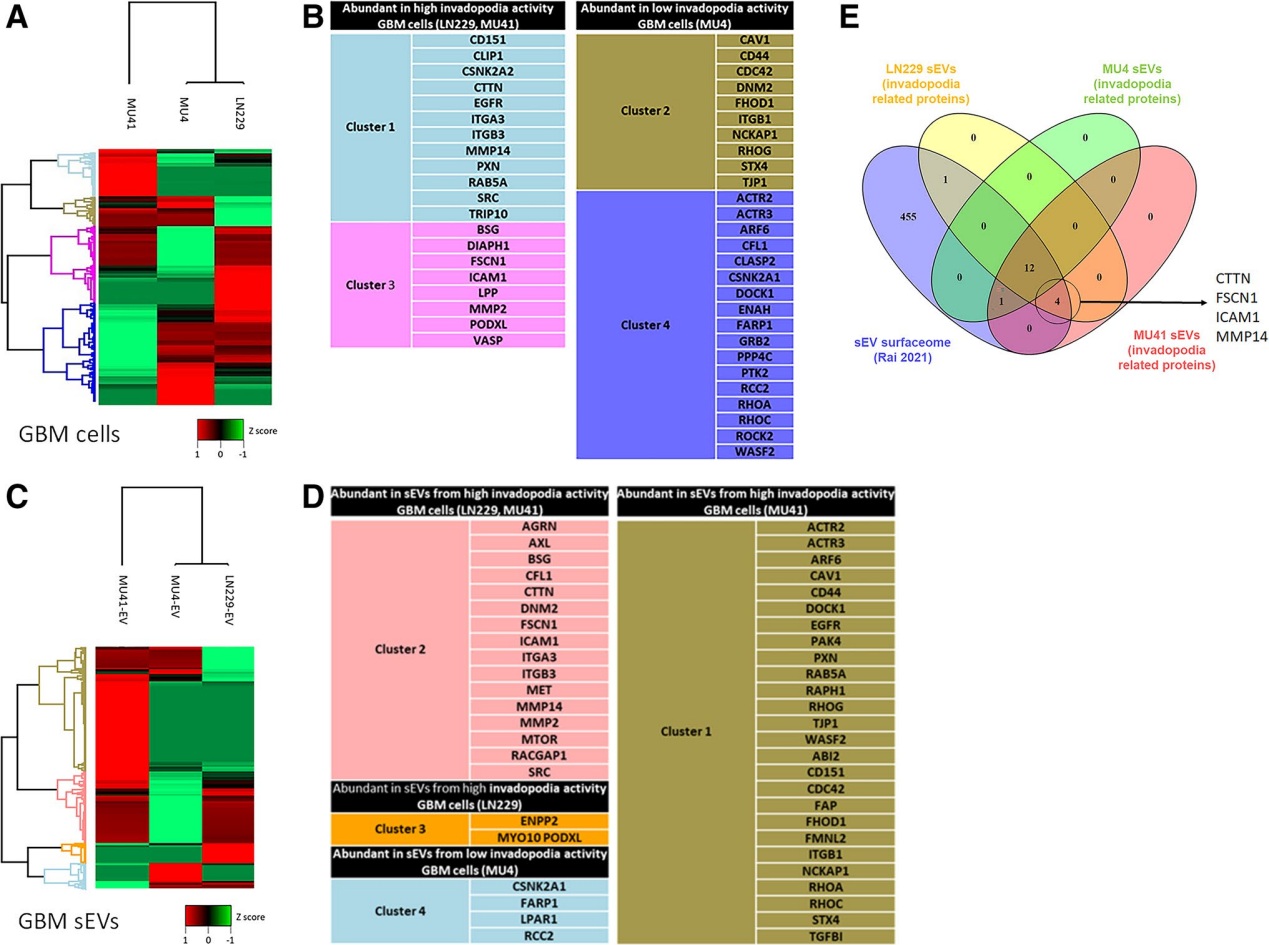
GBM cell line and sEV proteomes contain invadopodia related proteins. **(A**) Differentially expressed proteins detected in the GBM cell proteomes shown as a heatmap. Normalized Z-scores were generated using the Perseus Bioinformatics Platform and proteins into clusters based on hierarchical clustering. **(B)** Invadopodia-related proteins identified within each of the four coloured clusters that are shown in Fig. 3A. **(C)** Differentially expressed proteins detected in the sEVs from each GBM cell line shown as a heatmap based on hierarchical clustering. **(D)** Invadopodia-related proteins identified within each of the four clusters. **(E)** Venn diagram of the invadopodia-related proteins identified as part sEV ‘surfaceome’ upon comparison of sEV proteomes with the EV surfaceome identified by Rai et al. [43]. 12 invadopodia-related surface proteins were common to sEVs from all three cell lines, whilst 4 invadopodia-related surface proteins were identified exclusively in sEVs from LN229 and MU41 cells

The spatial gene expression profile in GBM cells and sEVs (Figs. 1, 2) of the invadopodia related proteins identified in their proteomes (Fig. 3) was examined using the Ivy GAP Glioblastoma Atlas [33]. An overview of the normalized gene expression z-score for each corresponding invadopodia related gene in GBM biopsies sampled from histologically distinct anatomic features designated as ‘Leading Edge’ and ‘Infiltrating Tumour’ is displayed in a heatmap. In addition, the spatial gene expression profile for the top 25 upregulated proteins identified in the GBM cell line proteome after RT/TMZ treatment was examined.

### Lipophilic dye labelling of sEVs and uptake assay

sEVs (500 μg/ml in PBS) from LN229 cells were labelled with 1 μM DiI (Invitrogen) and excess unbound dye removed by washing the labelled sEV pellet with sterile PBS via ultracentrifugation at 100,000xg (2 × 90 min). Control DiI samples were prepared in the absence of a sEV pellet to demonstrate non-aggregate dye formation. MU4 cells were incubated with 5 μg/ml of labelled sEVs, or an equivalent volume of control, for 4 h in serum-free OptiMEM (Thermofisher Scientific). Cells were washed with sterile PBS and fixed with 4% paraformaldehyde. Cell nuclei were stained with Hoechst (1:3000) for 10 min and images were acquired with a Leica Sp8 Lightning confocal microscope and analysed using ImageJ (Version 1.53a). N = 2.

### miRNA expression profiling

miRNA expression analysis was performed using a Nanostring nCounter Human V3 miRNA Array (Nanostring Technologies®). Briefly, MU4 GBM cells were incubated with LN229 cell-derived sEVs (25 μg/ml protein concentration) for 24 h (a corresponding MU4 GBM cell control was also prepared in the absence of LN229 sEVs). Cells were then washed with sterile PBS and incubated in serum-free OptiMEM (Thermofisher Scientific) for an additional 24 h. RNA was extracted using a RNeasy plus mini kit (Qiagen) according to the manufacturer’s protocol and 100 ng RNA was prepared for nCounter miRNA expression profiling according to the manufacturer’s recommendations (Nanostring Technologies®). The miRNA arrays were processed in an nCounter FLEX analysis system to generate RCC data files required for downstream analysis. Sample inputs were normalised to internal probes for housekeeping genes, as well as positive and negative controls, and analysed using the nSolver v 4.0 software platform. Only miRNAs above a normalized detection threshold of 100 transcript counts were included in the subsequent analyses. N = 1.

### RT/TMZ treatment

GBM cells (seeded 24 h prior at either 1 × 10^4^ cells per well in 96-well plates, or 2 × 10^5^ cells per well in 6-well plates) were treated with 2 Gy irradiation and incubated for 4 h (37 °C). Cells were then treated with 50 µM TMZ in serum-free OptiMEM for a further 24 h prior to the inclusion in functional assays, as performed previously [14].

### Cell viability assay

1 × 10^4^ GBM cells were seeded per well in 96-well plates in triplicate and incubated overnight at 37 °C. The cells were subsequently treated with 2 Gy irradiation and 50 µM TMZ on one, two or three consecutive days, and further incubated for one week before assessing the impact on cell viability using a CellTiter 96® Non-Radioactive Cell Proliferation Assay (‘MTT assay’, Promega) as per manufacturer’s instructions. Absorbance was measured at 570 nm using a Thermo electron Multiskan EX spectrophotometer, to determine the number of metabolically active cells. N = 3.

### Scratch wound closure migration assay

GBM cells were incubated with LN229-sEVs (25 µg/ml protein) in serum-free OptiMEM for 24 h, after which mitomycin C (final concentration – 5 μg/ml) was added 2 h prior to the introduction of a scratch wound in the confluent monolayer using a p1000 pipette tip. Wound closure was monitored over 24 h and images acquired using an Olympus IX50 microscope (4 × objective) at 0 h, 6 h and 24 h. Images were analysed using Image J (Version 1.53a) to define the area of wound closure relative to 0 h. N = 3.

### DMA treatment

Cells were treated with varying concentrations (0, 50 µM or 100 µM) dimethyl amiloride (DMA) in serum-free OptiMEM in triplicate wells of a 6-well plate for 24 h. Conditioned media were then centrifuged (2000xg for 15 min, 3166xg for 15 min, 10,000xg for 90 min) to remove cell debris and subjected to NTA analysis. FITC-gelatin degradation assays were conducted with LN229 GBM cells, as these cells have a high basal level of invadopodia-mediated degradation activity, and were pre-treated with increasing concentrations of DMA (0, 25 µM, 50 µM and 100 µM) for 24 h prior to seeding on FITC-gelatin.

### Vinorelbine tartrate treatment

Cells were treated with 1 µM vinorelbine tartrate (VT) in triplicate (either alone or in combination with 2 Gy RT/50 µM TMZ). After a 24 h incubation, cells were washed with sterile PBS after which the medium was replaced with serum-free OptiMEM and incubated for a further 24 h prior to NTA analysis to examine the effect on sEV secretion/particle number.

### 3D invasion assay

GBM cells were incubated with LN229-sEVs (25 μg/ml protein), RT/TMZ or RT/TMZ + VT as previously mentioned and then seeded in 24-well Cultrex BME Cell Invasion plates at 2.5 × 10^4^ cells/well for a period of 24 h and processed as per manufacturer’s instructions to quantify the invasive capacity of the cells.

### Statistical analysis

Statistical analyses were performed using an unpaired, two-tail Student’s t-test. Datasets were generated using the program GraphPad Prism 8 (GraphPad Software, CA, U.S.A), and represent mean ± SD. A probability value (*p* value) of less than 0.05 was considered statistically significant and indicated using the following asterisks: *p < 0.05, **p < 0.01, ***p < 0.001.

## Results

### GBM cells form functional invadopodia and secrete sEVs containing MMP-2

The ability of GBM cell lines U87MG, LN229, MU4 and MU41 to degrade FITC labelled-gelatin relative to cell number was assessed. We found that LN229 cells exhibited the highest gelatin-degrading activity (Fig. 1A, B). The number of invadopodia (indicated by rhodamine phalloidin–stained actin puncta) formed by each GBM cell line was measured, revealing that LN229 cells formed the highest number and the most active invadopodia per cell. Whilst MU4 cells exhibited a high actin puncta formation, the puncta were largely non-degradative (Fig. 1C). Zymographic analysis of GBM cell conditioned media revealed that MMP-2 (in both inactive (latent) and active forms) was the main protease secreted by the GBM cell lines, consistent with its role in GBM invasion and progression [34] (Fig. 1D), and correlated with the differences in FITC-gelatin degradation observed for each GBM cell model. No MMP-9 activity was detected in the GBM cell line conditioned media. sEVs were harvested from GBM cell conditioned serum-free media by differential ultracentrifugation and were characterised according to the minimal experimental requirements for EVs which include particle diameter, morphology, size distribution and EV-marker enrichment, as defined by the International Society for Extracellular Vesicles [22]. Nanosight Tracking Analysis (NTA) revealed that all GBM cells secreted similar quantities of sEVs, primarily with diameters < 200 nm (mean U87MG 106 nm, LN229 109 nm, MU4 102 nm, MU41 136 nm) (Fig. 1E). Representative NTA profiles for each GBM cell line are shown in Fig. 1F and characteristic vesicular morphologies revealed by cryo-EM, confirming a sEV size range of 40–200 nm for each GBM cell model are shown in Fig. 1G. Western blot analysis showed that sEVs in comparison to donor cells were enriched in the canonical EV marker, ESCRT-associated protein ALIX, and were absent in the EV-negative control, i.e., ER-associated protein Calnexin (Fig. 1H). Zymographic analysis of sEVs revealed MMP-2 (Fig. 1I). Relative to the other three GBM cell lines, reduced MMP-2 levels were present in MU4 derived sEVs, which also correlated with the lowest invadopodia-mediated FITC-gelatin degrading activity displayed by this GBM cell model.

### Proteomic analysis of GBM cell line derived sEVs reveals the presence of invadopodia-related protein cargo

Multiple proteins have been implicated in invadopodia formation and activity in various cancer cell lines. However, this has not been extensively studied in GBM cells and their secreted EVs. Thus, a comprehensive proteomic analysis of GBM cells and their derived sEVs was performed to determine their proteome landscape and to provide molecular insight of their invadopodia. The U87MG GBM cell line has been used for many GBM cell-based EV studies, with several groups performing proteomic analyses of the U87MG sEV cargo [35–39]. LN229 GBM cell line EVs have also been included in similar proteomic analyses, but these are limited compared to those of the U87MG GBM cell line, with a significant proportion only published within the last 3 years [40–42]. As such, we focussed our analysis on the three other GBM cell lines, which include two patient-derived cell lines, MU4 and MU41.

A total of 2354 and 2086 proteins were identified in the GBM cell and sEV proteomes, respectively (Fig. 2A, B). Several proteins that are often utilized as ‘EV markers’ were enriched in the sEVs compared to the donor GBM cells (Fig. 2C). We also identified abundant EV marker proteins across each GBM cell model and a combination of all models in this study. A comparative analysis of the GBM cell models with EV compendium Vesiclepedia, identified the cytoskeletal proteins (ACTB, ACTN1, ACTN4, CFL1), transmembrane proteins (BSG, ITGB1) and GTPases (RAB10, RAB7A, RHOA, RAC1) (Fig. 2D). Additionally, 244 sEV proteins common to sEVs from all three GBM cell lines were identified as surface proteins upon comparison with EV surfaceome recently reported by Rai et al. [43], including adhesion-related proteins (ITGA3, ITGA5, ITGA6, ITGAV, ITGB1, NPTN), cytoskeletal proteins (ACTR2, ACTR3, CFL1) and growth factors (e.g. EGF), known to be involved in cancer cell growth and invasion (Fig. 2E). As the GBM cell lines exhibited varying levels of invadopodia-mediated FITC-gelatin degrading activity, we examined their proteomes and identified distinct clusters of proteins with differential expression patterns across the three GBM cell lines (Fig. 3A) and in their corresponding sEV proteomes (Fig. 3C). Further interrogation of these clusters revealed that GBM cell lines with high invadopodia matrix-degrading activity (LN229 and MU41) had a greater abundance of proteins involved in invadopodia maturation and proteolytic activity (including BSG, CLIP1, MMP14, MMP2, RAB5A) than the low invadopodia activity cell line, MU4 (Fig. 3B). Furthermore, these high invadopodia activity cell lines secreted sEVs with a greater abundance of proteins involved in the regulation of invadopodia formation (CTTN, CFL1, SRC, ITGA3, ITGB3 – Cluster 2) and proteolytic activity (MMP2, MMP14, BSG/CD147 – Cluster 2) (Fig. 3D).

As the sEV surface proteome (surfaceome) dictates the ability of sEVs to interact with their environment, we next compared the invadopodia-related proteins identified in GBM sEVs to the EV surfaceome (recently reported by Rai [43]). A total of 18 invadopodia-related proteins were identified as sEV surface components, including adhesion molecules that may interact with receptors on target GBM cells (ITGA1, ITGA3, ICAM1) and proteolytic proteins (BSG, MMP14) that may activate extracellular MMP-2 to promote ECM degradation (Fig. 3E). Additionally, we found that four of these sEV surface proteins (CTTN, FSCN1, ICAM1, MMP14) were exclusively present in sEVs from high invadopodia activity cells, which have previously been reported in sEVs that are preferentially secreted from invadopodia in breast cancer cells [44]. This further substantiates a link between invadopodia and sEVs and identifies components of the sEV surfaceome that may directly interact with recipient GBM cells to promote invadopodia activity.

### GBM cells are reprogrammed to promote invadopodia following sEV transfer

**Fig. 4:**
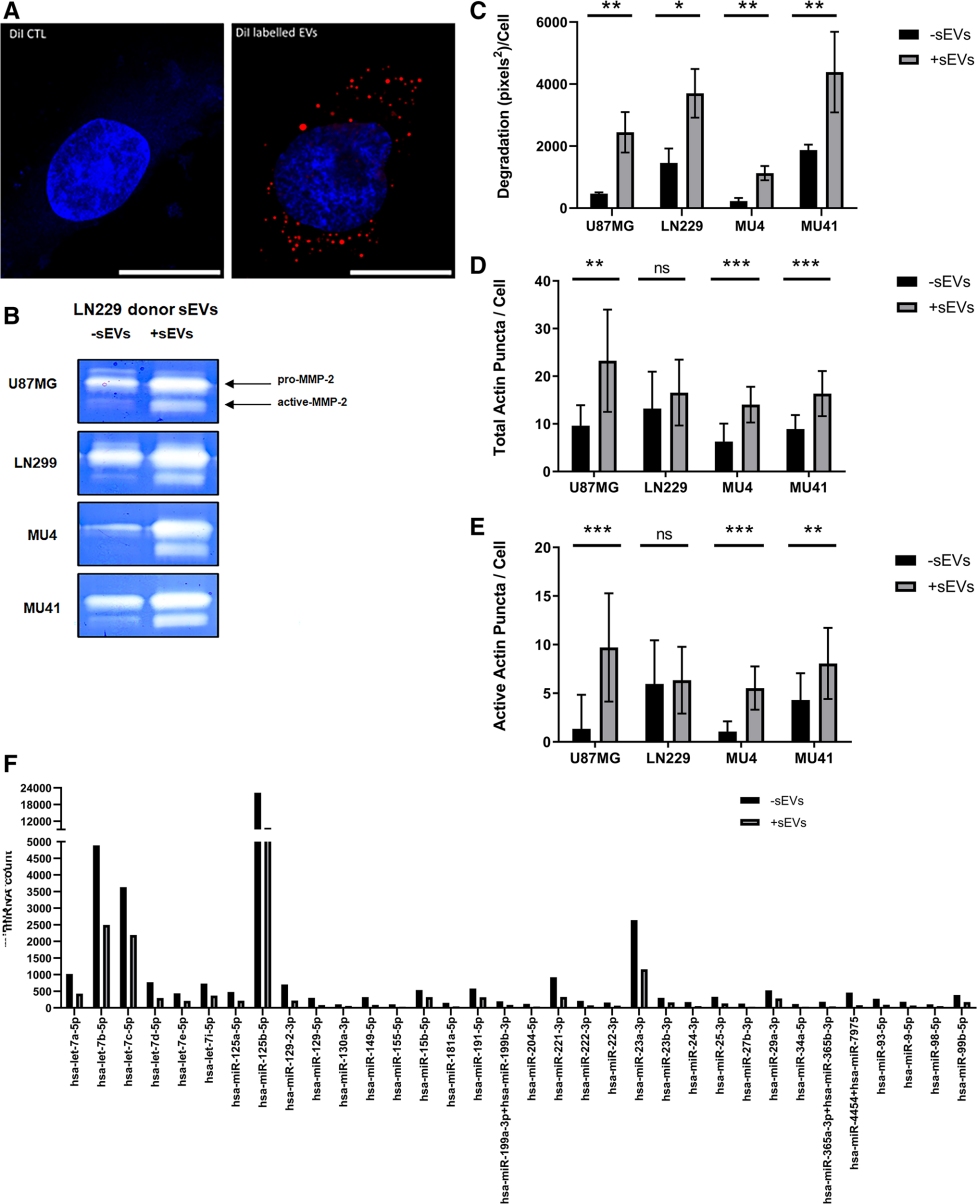
GBM cells are reprogrammed to promote invadopodia following sEV transfer. **(A)** Representative confocal image of MU4 GBM cells incubated (4 h) with 5 μg/ml Dil-labelled LN229 sEVs (DiI labelled EVs) or equivalent amounts of a DiI background control (DiI CTL). Internalized sEVs are visualized as fluorescent red dots. Cell nuclei were stained with Hoechst (blue). Scale bar (10 μM) n = 2. **(B)** Zymographic analysis indicating increased MMP-2 secretion from GBM cells pre-incubated in serum-free OptiMEM in the absence (-sEVs) or presence (+ sEVs) of LN229 sEVs for 24 h. Graphical representation of observed changes in **(C)** FITC-gelatin degradation, **(D)** total actin puncta per cell and **(E)** active actin puncta per cell in the absence (-sEVs) or presence (+ sEVs) of a pre-incubation with LN229-cell line derived sEVs. Following pre-incubation with LN229 sEVs, the culture medium was removed, the cells were washed with PBS and cell culture medium (without LN229 sEVs) was added to the cells for an additional 24 h to conduct the invadopodia assay. (n = 3 experiments; mean ± SD *p < 0.05; **p < 0.01, ***p < 0.001, unpaired two-tailed student’s t-test). **(F)** Quantification of miRNA expression utilizing a Nanostring® nCounter Human V3 miRNA array examining the impact of LN229 sEVs in recipient MU4 cells. miRNAs above a normalized detection threshold of 100 transcript counts were included in the analysis with 34 miRNAs demonstrating a > 1.5-fold decrease. (MU4 cells incubated with LN229 sEVs (+ sEVs); MU4 cells incubated without LN229 sEVs (-sEVs)

As invadopodia-related proteins are contained in the sEV cargo across distinct cell models, we next determined if the transfer of sEV cargo between GBM cells can promote invadopodia formation and activity. Recipient GBM cells were incubated with sEVs from the high invadopodia-activity donor cell line LN229. DiI-labelled sEVs were utilized to confirm interaction with recipient cells (Fig. 4A). Following incubation with the LN229-derived sEVs, recipient GBM cells exhibited an increase in MMP-2 secretion and activity (Fig. 4B), FITC-gelatin degradation (Fig. 4C), and invadopodia formation and activity (Fig. 4D, E).

Assessment of the impact of sEV cargo transfer at the miRNA level in recipient GBM cells using a Nanostring nCounter Human V3 miRNA array, revealed a significant decrease in the expression of 34 miRNAs in MU4 cells incubated with LN229 sEVs (Fig. 4F). By utilizing the MirTarBase and miRDB databases, we found that several of the miRNA target genes are involved in invadopodia formation and activity, including MMP-2, MMP-9, Grb2, SH3PXD2A (TkS5), WASL (N-WASp) and Src (Table S1). Together, these results indicate that sEVs from donor GBM cells with a high invadopodia activity may reprogram recipient GBM cells to promote a pro-invadopodia phenotype.

### RT/TMZ treatment promotes a pro-invadopodia phenotype in GBM cells

**Fig. 5:**
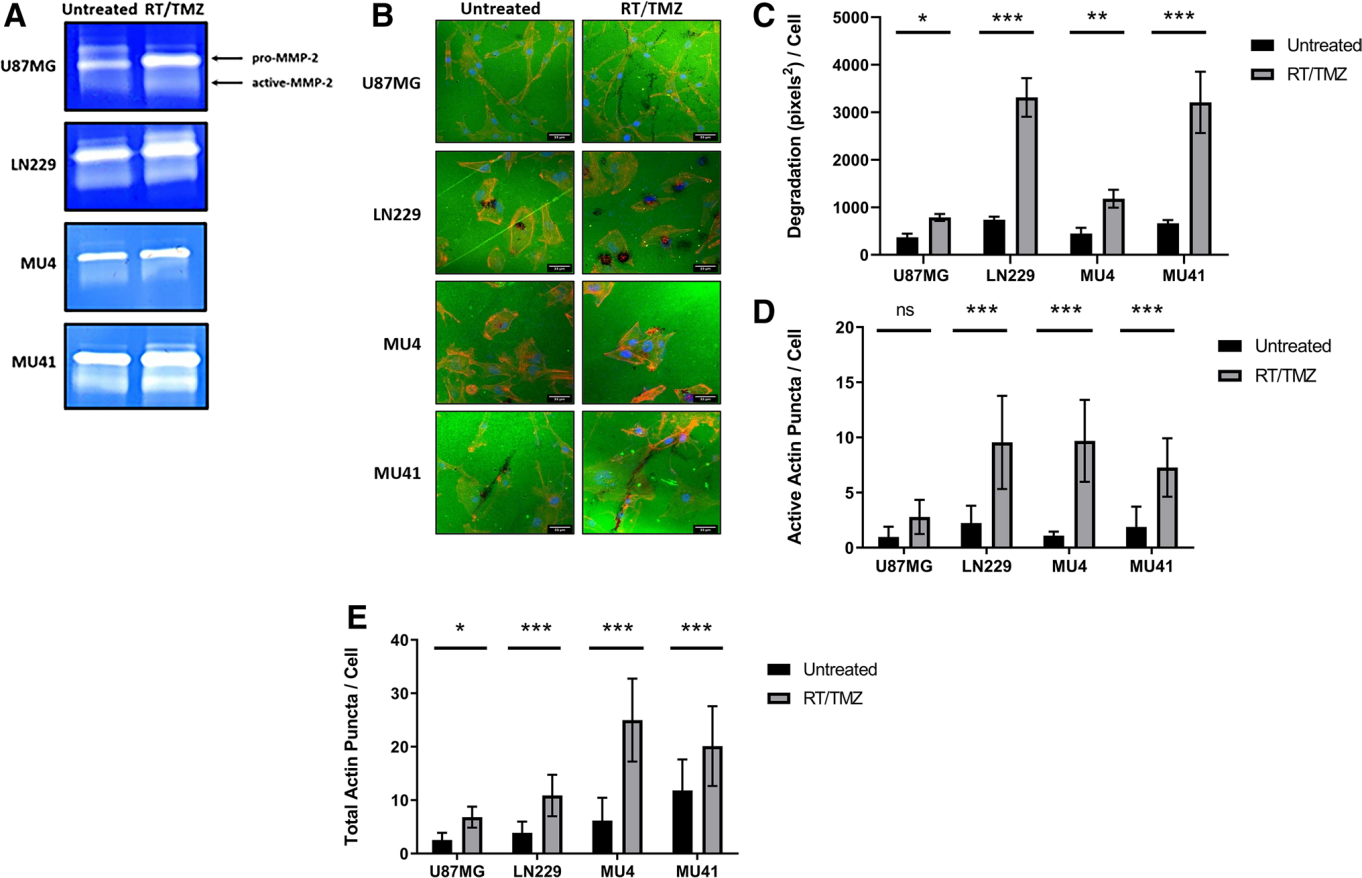
Invadopodia formation/activity is enhanced in GBM cells treated with RT/TMZ. **(A)** Zymographic analysis of conditioned serum-free OptiMEM.® medium 24 h post-RT/TMZ treatment showing increased MMP-2 secretion from the GBM cells in response to treatment. **(B)** Representative confocal images of untreated or RT/TMZ treated GBM cells seeded on FITC-labelled gelatin for 24 h (Scale bar, 33 μm). Graphical representation of observed increases in **(C)** FITC-gelatin degradation, **(D)** total actin puncta per cell and **(E)** active actin puncta per cell in response to RT/TMZ treatment of the GBM cells. (n = 3 experiments; Mean ± SD, *p < 0.05, ***p < *0.001*, NS = non-significant; unpaired two-tailed student’s test)

Previous studies have shown that GBM cells which survive RT/TMZ treatment may exhibit an enhanced invasive phenotype [5–9]. Therefore, we next examined whether an increase in invadopodia activity contributes to this effect. We found that GBM cells treated with RT (2 Gy) and TMZ (50 μM), corresponding to the TMZ concentration range (5.15 – 51.5 μM) in the cerebrospinal fluid (CSF) of GBM patients [45], displayed an increase in MMP-2 secretion (Fig. 5A), invadopodia-mediated FITC-gelatin degradation (Fig. 5B) and invadopodia formation (Fig. 5D).

**Fig. 6:**
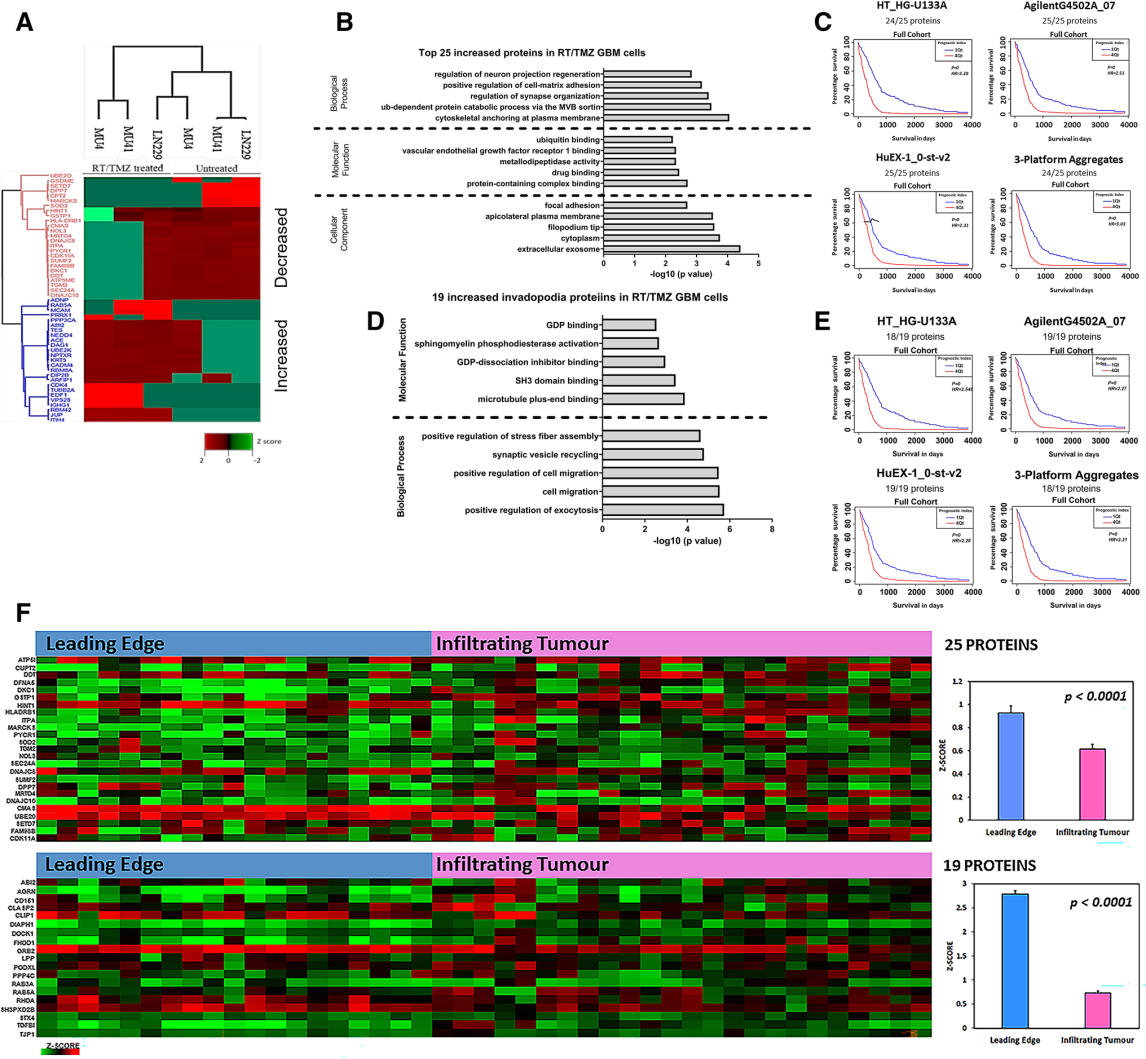
Invasion and invadopodia-related proteins are increased in GBM cells following RT/TMZ treatment. **(A)** The top 50 differentially expressed ( ±) proteins in RT/TMZ treated GBM cells displayed as a heatmap (determined by the average log2-transformed LFQ ratios of untreated versus RT/TMZ treated GBM cells and normalized Z-score). **(B)** Functional enrichment analysis of the top 25 increased proteins detected in RT/TMZ treated GBM cells (top 5 annotations with the most significant *p* values calculated by a two-sided hypergeometric test are shown). **(C)** Correlation of mRNA expression levels corresponding to the top 25 increased proteins and GBM patient survival interrogated in the Glioblastoma Bio Discovery Portal (GBM-BioDP). **(D)** Functional enrichment analysis of the 19 invadopodia-related proteins with increased expression in RT/TMZ treated GBM cells (top 5 annotations with the most significant *p* values calculated by a two-sided hypergeometric test are shown). **(E)** Correlation of mRNA expression levels corresponding to the 19 increased invadopodia proteins and GBM patient survival interrogated in the Glioblastoma Bio Discovery Portal (GBM-BioDP). *P* values below *p* = 0.01 are listed as ‘p-val = 0’. **(F)** Normalized RNA sequencing data from the IVY GAP database showing expression of the corresponding genes for the top 25 increased proteins and 19 increased invadopodia-related proteins detected in the GBM cell lines post-RT/TMZ treatment in histologically distinct anatomic regions, i.e., leading edge and infiltrating tumour, displayed in a heatmap. Positive expression Z-scores for each gene within these regions revealed an overall increased expression in the ‘Leading Edge’ of the tumour

Examination of differentially expressed (DE) proteins in the proteome of GBM cells after RT/TMZ treatment revealed an increase in the abundance of 540 proteins and a decrease in 401 proteins across all three GBM cell lines. To understand the proteome composition that is implicated in the enhanced pro-invadopodia phenotype displayed by GBM cells following exposure to RT/TMZ treatment, the top 25 significantly increased proteins common to all GBM cell lines post-treatment were investigated (Fig. 6A). Functional enrichment analysis revealed that these upregulated proteins were associated with invadopodia-related cellular components (including ‘filopodium tip’ and ‘focal adhesions’), as well as sEVs including exosomes (incorporating ‘the MVB sorting pathway’ and ‘cytoskeletal anchoring at plasma membrane’) (Fig. 6B), indicating that the sEV fraction isolated by ultracentrifugation contains MVB-derived exosomes as well as surface derived vesicles. In addition, we found that high mRNA expression levels corresponding to these 25 increased proteins correlated with shorter GBM patient survival times, suggesting that the increased expression of these proteins in RT/TMZ treated GBM cells may be of prognostic significance, when evaluating the database as a full cohort (classical, mesenchymal, proneural and neural GBM subtypes) (Fig. 6C). Supporting a pro-invasive phenotype after RT/TMZ treatment, 11 of these proteins are known to promote invasion, however only eight of these have been reported to be involved in GBM tumour cell invasion (Table S2). The invasion genes that were evaluated using the ‘Full Cohort’ of GBM-BioDip incorporating all GBM subtypes, were also analysed using the separate GBM subtypes (Fig. S5A). The summary of the analyses across the different platforms and GBM subtypes indicates that an increased impact on survival can occur across the classical, mesenchymal and proneural subtypes.

The abundance of 19 previously reported invadopodia-related proteins was also found to be increased in GBM cells following RT/TMZ treatment (Table S3), and were primarily found to be involved in exocytosis and microtubule-mediated vesicle trafficking, suggesting an increase in the transport of vesicles to invadopodia after RT/TMZ treatment (Fig. 6D). High mRNA expression levels corresponding to these invadopodia-related proteins were also found to correlate with shorter GBM patient survival times (Fig. 6E). Again, we performed analyses using the separate GBM subtypes (Supp. Fig S5B) and found that an increased impact on survival can occur across all four subtypes based upon the invadopodia-related gene expression. Importantly, we found that the corresponding genes of the top 25 increased proteins and the 19 invadopodia proteins increased after RT/TMZ treatment were highly expressed at the leading edge of primary GBM tumours (Fig. 6F), where tumour cells can form invadopodia to degrade the ECM, thereby facilitating invasion into the surrounding healthy brain parenchyma.

### RT/TMZ treatment alters sEV composition and secretion

**Fig. 7:**
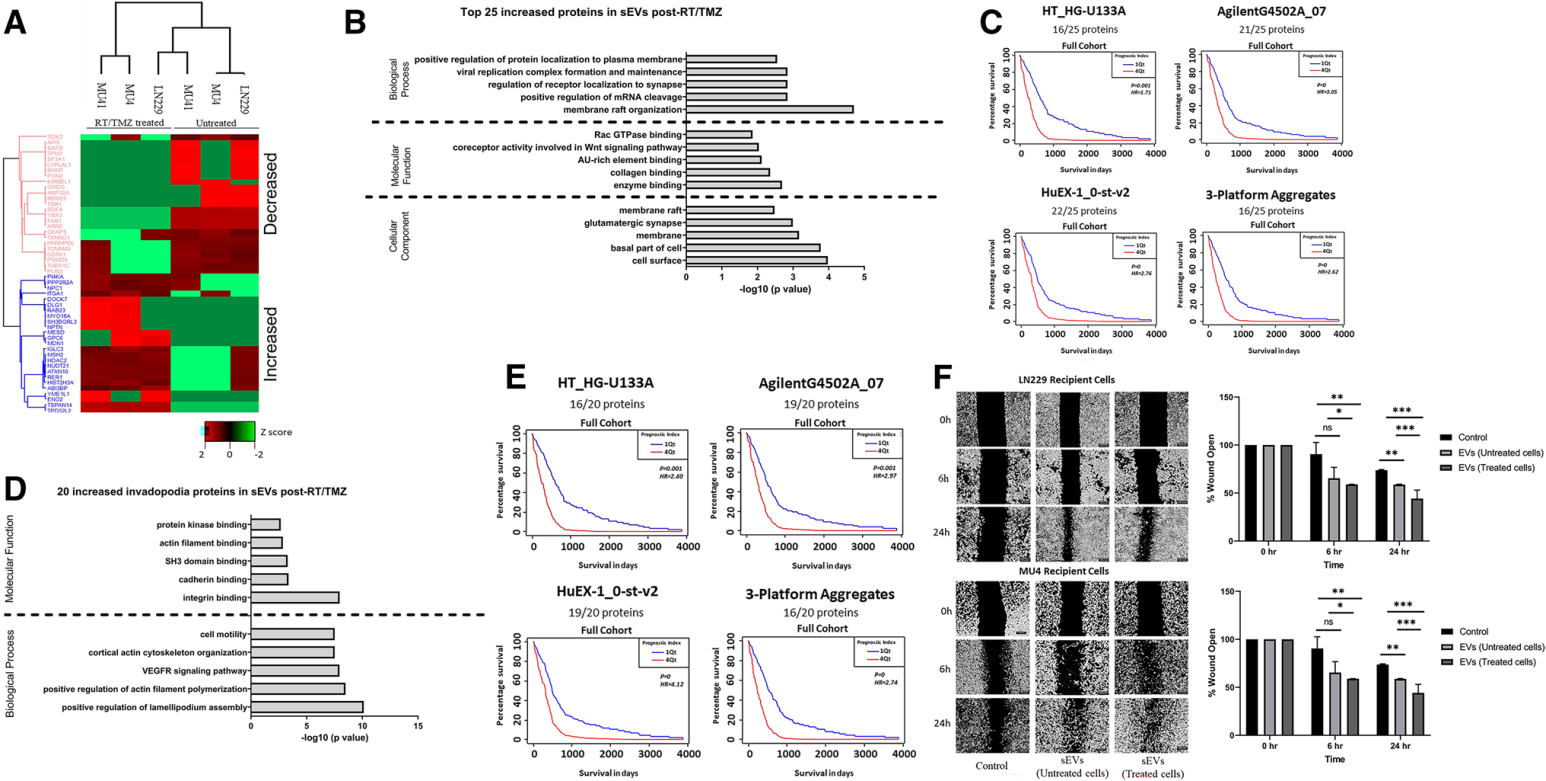
RT/TMZ treatment alters the composition of GBM cell line-derived sEVs. **(A)** Top 50 differentially expressed proteins detected in the sEVs harvested from RT/TMZ treated GBM cell lines displayed as a heatmap (normalized Z-score). **(B)** Functional enrichment analysis of the top 25 increased proteins detected in sEVs harvested from RT/TMZ treated GBM cells (top 5 annotations with the most significant *p* values calculated by a two-sided hypergeometric test). **(C)** Correlation of mRNA expression levels corresponding to the top 25 increased proteins and GBM patient survival interrogated in the Glioblastoma Bio Discovery Portal (GBM-BioDP). **(D)** Functional enrichment analysis of the 20 invadopodia-related proteins with increased expression in sEVs from RT/TMZ treated GBM cells (top 5 annotations with the most significant *p* values calculated by a two-sided hypergeometric test). **(E)** Correlation of mRNA expression levels corresponding to the 20 increased invadopodia proteins and GBM patient survival data interrogated in the Glioblastoma Bio Discovery Portal (GBM-BioDP). *P* values below *p* = 0.01 are listed as ‘p-val = 0’. **(F)** sEVs harvested from untreated and RT/TMZ treated LN229 cells were added to recipient LN229 and MU4 cells (protein—25 μg/ml) after which the impact on GBM cell migration was assessed using a scratch wound closure assay (n = 3 experiments; 4 images per cell line per experiment; mean ± SD, *p < 0.05; **p < 0.01; ***p < 0.001, non-significant = ns, unpaired two-tailed student’s test)

Next, the impact of RT/TMZ treatment on the composition of sEVs was examined. We found that 676 proteins were significantly increased and 488 proteins were decreased in the sEV proteome following RT/TMZ treatment. Identification of the top 25 increased DE sEV proteins following RT/TMZ treatment (Fig. 7A) revealed functional associations to collagen-, enzyme- and receptor-binding, suggesting roles in mediating sEV interactions with ECM components and/or receptors on recipient cells (Fig. 7B). Furthermore, high mRNA expression levels corresponding to these increased sEV proteins were found to correlate with a poorer GBM patient survival (Fig. 7C), when examined as a full cohort encompassing all four GBM subtypes. However, when evaluated within the separate GBM subtypes, the summary of the analyses (Fig S5C) indicates that an increased risk of impact on survival can occur across the classical, mesenchymal and proneural subtypes. Importantly, fourteen of these proteins are known to promote invasion, whilst only 11 have been previously reported in GBM (Table S2).

The abundance of 20 established invadopodia-related proteins was also increased in the proteome of sEV from one or more GBM cell lines after RT/TMZ treatment. These have largely been shown to exhibit functional associations with signalling pathways known to drive actin polymerisation (‘integrin binding’, ‘SH3 domain binding’), suggesting a role in promoting invadopodia initiation in recipient cells (Fig. 7D, Table S3). Notably, whilst MU4 sEVs initially lacked FSCN1 and MMP14 in their surfaceome, these two proteins were identified in MU4 sEVs following RT/TMZ treatment, suggesting that these sEVs may exhibit enhanced invadopodia-promoting activity upon interaction with recipient GBM cells. High mRNA expression levels corresponding to the increased invadopodia-related proteins in sEVs after RT/TMZ treatment examined within the ‘Full Cohort’ were found to correlate with shorter GBM patient survival times (Fig. 7E). Importantly, when examining these proteins using the individual GBM subtypes we found that they exhibit an increased risk of impact on survival in classical, mesenchymal and proneural cases (Fig S5D). Also, whilst incubation with sEVs from the high invadopodia activity donor cell line LN229 increased recipient GBM cell migration rates, a further enhancement in migration rate was observed with sEVs harvested from LN229 GBM cells after RT/TMZ treatment (Fig. 7F).

We next examined whether sEV secretion was impacted by RT/TMZ treatment. A significant increase in sEV secretion was observed from GBM cells following a single RT/TMZ treatment (Fig. S1), whilst the sEV size distribution remained unchanged (data not shown). These results support previous studies reporting an increase in sEV secretion from irradiated U87MG cells, but these studies did not investigate the impact of RT in combination with TMZ [46, 47]. Therefore, we investigated whether the changes observed in sEV secretion were primarily due to RT, TMZ or a combination of both. Whilst all treatment groups showed an enhanced sEV secretion compared to untreated cells, the most significant increase in sEV secretion was consistently observed with a combination of RT and TMZ treatment (Fig. S1). The relevance of increased sEV secretion in GBM was further supported by the observation that elevated expression of canonical EV markers (ALIX, CD9, CD63, CD81, CD151 and TSG101) correlated with a poorer GBM patient survival (Fig. S1). Importantly, RT/TMZ treatment resulted in both an increase in invadopodia activity and in secretion of sEV.

### Therapeutic regulation of invadopodia activity alters sEV secretion

**Fig. 8:**
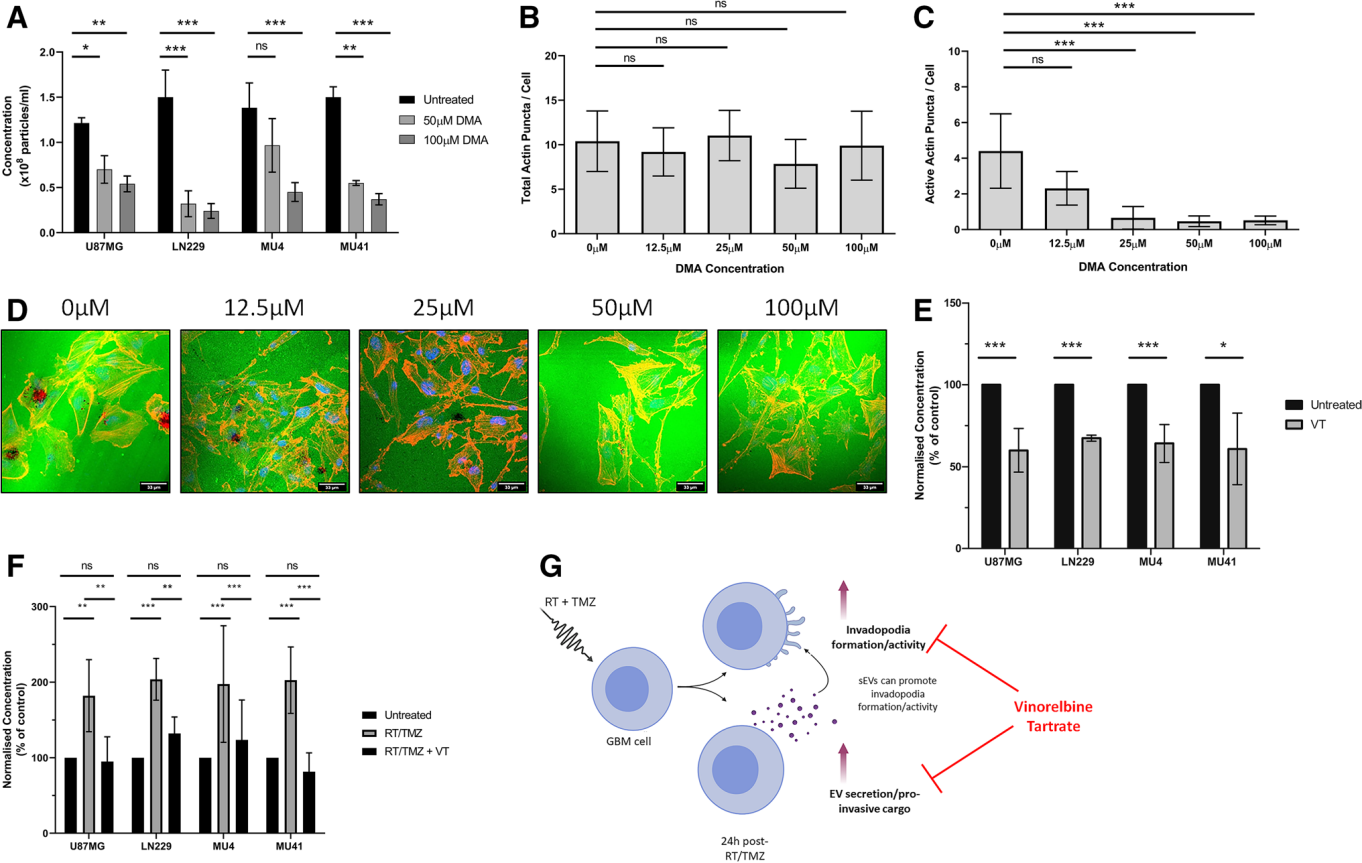
Therapeutic regulation of invadopodia alters sEV secretion. **(A)** Increasing concentrations of DMA (50 and 100 μM) reduces sEV secretion relative to the untreated control. sEV concentrations were normalized to 1 × 10^5^ cells and triplicate readings were acquired (n = 3 experiments; mean ± SD, *p* < 0.05, **p < 0.01, ***p < 0.001 non-significant = ns, unpaired two-tailed student’s test). **(B)** Increasing concentrations of DMA (12.5, 25, 50 and 100 μM) did not impact the total number of invadopodia per cell, but **(C and D)** significantly reduced invadopodia-mediated FITC-labelled gelatin degradation in LN229 GBM cells. Scale bar, 33 μm. **(E)** GBM cells were treated with 1 μM VT for 24 h before a subsequent 24 h incubation in serum free OptiMEM medium prior to NTA analysis. VT treated GBM cells display a reduced sEV secretion compared to untreated control cells. **(F)** NTA analysis of GBM cell lines either untreated or treated with RT/TMZ (2 Gy, 50 μM- 24 h) or RT/TMZ + 1 μM VT (2 Gy, 50 μM, 1 μM VT – 24 h), indicating that VT can reduce the enhanced sEV secretion observed after RT/TMZ treatment. **(G)** A schematic overview of the experimental findings outlining that VT treatment can reduce the observed enhanced invadopodia activity and sEV secretion from GBM cells post-RT/TMZ treatment

As invadopodia activity in GBM cells may be regulated via sEVs, novel therapeutic strategies that target both invadopodia activity and GBM-derived sEV-mediated function may lead to improved outcomes for GBM patients. Dimethyl amiloride (DMA) is an ion channel blocker that is used to reduce sEV secretion triggered by an increase in intracellular Ca^2+^ [48], and this has also been reported to enhance invadopodia-related ECM degradation [49]. We found that DMA treatment reduced sEV secretion from GBM cells in a concentration-dependent manner (Fig. 8A) and, although there was no change in invadopodia formation (Fig. 8B), a reduction in invadopodia-mediated FITC-gelatin degrading activity was observed (Fig. 8C, D), indicating that sEV secretion and the ECM-degrading activity of invadopodia may be linked. These findings support the hypothesis that invadopodia serve as additional sites of sEV release, as previously proposed in a study investigating EV/exosome release from head and neck squamous cell carcinoma cells [50].

As the proteomic analysis of RT/TMZ treated GBM cells revealed an increase in invadopodia-related proteins involved in microtubule-mediated vesicle trafficking (Fig. 6D), therapeutic agents that destabilise microtubules may impact both invadopodia-mediated ECM degradation and sEV secretion. In agreement with previous data from our laboratory showing that the microtubule-destabilising agent VT (an FDA approved agent for non-small cell lung cancer) reduces invadopodia activity in RT/TMZ treated GBM cells [14], here we found that 1 μM VT treatment of GBM cells results in a significant reduction of sEV secretion (Fig. 8E), without a significant loss in GBM cell viability (Fig. S3). Furthermore, the enhanced levels of sEV secretion observed after RT/TMZ treatment were also significantly reduced when VT was included as an adjunct treatment (Fig. 8F). This suggests that a disruption of microtubule dynamics, with a repurposed FDA-approved agent such as VT, may be a promising novel adjuvant therapeutic strategy to target invadopodia- and sEV-mediated invasion in GBM cells surviving RT/TMZ treatment (Fig. 8G). However, further insights at the molecular level are required to firmly establish the true impact of the enhanced EV secretion observed post-RT/TMZ treatment.

### 3D invasion of GBM cells is enhanced post RT/TMZ treatment or GBM cell-derived sEV incubation

As we have shown that incubation of GBM recipient cells with LN229-sEVs or treatment with RT/TMZ promotes invadopodia activity, we also examined whether the invasive capacity of the GBM cells through a matrix was impacted using a commercial invasion assay. We found that the invasive capacity of the GBM cells is indeed enhanced post-sEV incubation or RT/TMZ treatment (Fig. S4). In addition, we found that inclusion of vinorelbine tartrate with RT/TMZ treatment resulted in a reduction in the enhanced GBM cell invasion observed after RT/TMZ treatment alone.

## Discussion

A defining feature of GBM tumours is their high capacity for dissemination from the periphery of the tumour mass, allowing cells to escape surgery and treatment resistant tumour cell populations to persist in the brain, leading to tumour recurrence. In this study, we show that GBM cells form functional FITC-gelatin degrading invadopodia to facilitate tumour invasion and secrete sEVs that carry invadopodia-related cargo to promote invadopodia formation and activity in recipient GBM cells. Crucially, both invadopodia formation/activity and sEV secretion were enhanced following RT/TMZ treatment, providing novel insights into the response of GBM cells to standard therapy.

We found that GBM cells are reprogrammed to promote invadopodia formation and activity following incubation with sEVs from a high invadopodia activity donor cell line, LN229, indicating that sEV transfer between GBM cells may enhance tumour invasion. This notion was also supported by a increased invasion of GBM cells through a commercial extracellular matrix (ECM) after incubation with sEVs. Proteomic analysis revealed that these sEVs contained various cytosolic and membrane-bound surface proteins known to facilitate invadopodia formation/activity, including Src kinase and integrins α3/β1/β3, actin regulators (cortactin) and proteases (MMP-2 and MT1-MMP). This suggests that functional changes observed in GBM cells may result from the direct transfer of invadopodia-related sEV cargo proteins or via the binding of the sEV ‘surfaceome’ to receptors on the recipient cells to stimulate intercellular signalling cascades. For example, whilst integrins are traditionally known to stimulate invadopodia formation in response to ECM components [51], the same pathways may also be activated by binding to sEV surface proteins.

Interestingly, four invadopodia-related sEV surface proteins were exclusively identified in sEVs from high invadopodia activity GBM cell lines, and these are known to play important roles in proteolysis (MMP14; MT1-MMP), adhesion-related signalling cascades (ICAM1; intercellular adhesion molecule-1, and cytoskeletal regulation/cell motility (FSCN1; fascin, CTTN; cortactin). Cortactin and fascin have been implicated in endosomal trafficking to invadopodia in breast cancer cells, resulting in the release of sEVs that were enriched with invadopodial proteins such as MT1-MMP [44]. This supports the enrichment of these proteins on the surface of sEVs from high invadopodia activity GBM cells and indicates a potentially important role for these proteins in the horizontal communication between GBM cells. As surface proteins are able to capture sEVs from biofluids [52], these may warrant further investigation as potential biomarkers of invasive disease in GBM patients. Collectively, the data presented here provide valuable insights into how GBM cells utilize sEV transfer to induce the formation of functional invadopodia in recipient GBM cells. A schematic representation of the proposed interactions between sEVs and invadopodia in GBM cells is shown in Fig. S2.

Using an in vivo breast cancer model, Zomer et al. [53] found that less malignant T47D cells that take up MDA-MD-231 cell-derived EVs exhibit an enhanced migration, irrespective of T47D and MDA-MB-231 cells present in one subcutaneous tumour (local communication) or present as separate tumours in contralateral mammary glands (distant communication). However, they did find that the proximate presence of MDAMB-231 cells favoured a migration-inducing microenvironment for T47D cells when in close proximity. As activation of migration and invasion is one of the hallmarks of cancer [54], the transfer of various biomolecules can affect multiple parameters including the migratory potential of the cell. When T47D cells took up EVs from less migratory MCF-7 cells, a reduction in the metastatic potential of these breast cancer cells was observed. The transfer of metastatic capacity observed in our study is very likely due to the transfer of multiple specific/non-specific functional biomolecules that have been loaded into the EVs including DNA, miRNAs, mRNAs, proteins and lipids, impacting multiple migratory and metastasis related pathways, indicating that the transfer of EVs between tumour cells plays a role in tumour progression. So, acceleration of tumour progression may occur through the transfer of EVs from highly metastatic cells to less malignant cells. This notion is supported by studies showing correlations between EVs that are present in the tumour milieu and body fluids of cancer patients and tumour progression parameters such as cell survival, pro-angiogenic, immunosuppressive or pro-metastatic processes [55–57]. It has been found that the EV cargo from GBM cells differs from that of normal glial cells [58], including mutant oncoproteins, oncogenic transcripts and miRNAs [59] that promote tumour progression through the creation of a permissive environment.

Previous studies have also shown that GBM cells surviving RT and/or TMZ treatment may exhibit enhanced migratory and invasive abilities [5–9, 60], implying that the current therapeutic approach for GBM may have a counterproductive effect on surviving cells. Although these findings highlight a key role for invasion in response to the current treatment for GBM, few studies have addressed the combinatorial impact of both RT and TMZ. Through our approach of combining clinically relevant doses of RT (2 Gy) and TMZ (50 µM), we were able to show that the previously reported enhanced invasive capabilities of GBM cells post-RT/TMZ treatment may be attributed not only to the increased activities of invadopodia [12, 14], but may also be linked with sEV secretion, allowing the transfer of EV cargo between GBM cells. The increase in EV secretion is supported by previous studies showing that either RT or TMZ treatment, albeit at higher than clinical relevant doses, can influence EV secretion from GBM donor cells [46, 47, 61, 62]. Our data show that even at the lower clinical doses, the combination of RT and TMZ can promote EV secretion, in addition to promoting invadopodia activity.

Our proteomic analysis of the GBM cell proteome after RT/TMZ treatment supported the acquisition of a pro-invasive phenotype and highlighted key upregulated invadopodia-related proteins with prominent roles in microtubule-mediated vesicle transport, indicating that GBM cells (across various GBM cell models) may respond to RT/TMZ treatment by increasing the transport of vesicles to invadopodia. Analysis of the GBM and EV proteomes post-treatment and the corresponding survival data using the TCGA within GBM-BioDip revealed that the most highly expressed proteins result in an increased risk on survival across the classical, mesenchymal and proneural subtypes. Indeed, vesicle-mediated membrane trafficking has been found to be crucial for the delivery of MMPs to the maturing invadopodia in breast cancer cells [63, 64], and it has been proposed that invadopodia themselves can function as supplementary sites of EV release at the cell surface [50], supporting our observations of increased invadopodia formation and activity and sEV secretion in GBM cells after RT/TMZ treatment. In addition, we found that sEVs released by RT/TMZ treated GBM cells contained elevated levels of proteins known to drive invadopodia initiation, including the EV surface proteins fascin and MMP14 that were absent in sEVs secreted from untreated MU4 cells. This may result in a positive feedback loop, whereby increased invadopodia formation and sEV secretion maintain one another in a population of GBM cells as a response to RT/TMZ treatment. Consequently, this would lead to a prolonged invasive phenotype, contributing to tumour recurrence and treatment failure [50]. Importantly, we found that DMA treatment reduced both the capacity of GBM cells to secrete sEV and their invadopodia mediated FITC-gelatin degrading ability, suggesting that if there are fewer vesicles with pro-invasive cargo being secreted for recipient cells to utilize, a reduction in invadopodia activity will result.

In light of these findings, novel therapeutic strategies additional to RT/TMZ treatment that disrupt both invadopodia activity and sEV-mediated communication may limit the treatment induced invasive potential of GBM and improve patient survival. As our proteomic analysis of GBM cells revealed an upregulation of proteins involved in microtubule trafficking after RT/TMZ treatment, it may be suggested that by targeting microtubules, we may impede the activity of invadopodia and their capacity to act as additional sEV secretion sites. Vinorelbine tartrate (VT), a FDA approved agent for non-small cell lung cancer, belongs to the vinca alkaloid group of drugs that destabilise microtubules by preventing a phenomenon known as ‘treadmilling’ involving the addition of tubulin subunits at the positive end of lengthening microtubules [65]. This process assists in stabilization of the invadopodium core and is also involved in the transport of secretory vesicles, such as those containing MMPs, to the tip of the invadopodium for subsequent secretion and degradation of the surrounding ECM [51]. In accordance with previous data from our laboratory showing that VT reduces invadopodia-mediated FITC-gelatin degradation in GBM cells surviving RT/TMZ [14], we found that VT treatment reduced the increased sEV secretion from GBM cells post-RT/TMZ treatment. VT is less neurotoxic than other vinca alkaloids [66] and has been demonstrated to cross the blood–brain barrier in a preclinical mouse model of brain metastases of breast cancer, with a detectable concentration range between 0.5 µM and 7 µM in the brain metastases [67]. This is encouraging as we observed a significant reduction in sEV secretion from various GBM cell models when 1 μM VT was combined with RT/TMZ treatment. These data highlight the potential for VT to be utilized as a promising anti-invasive agent in combination with current therapy to target enhanced invadopodia activity and sEV secretion in RT/TMZ treated GBM. Previously, we have shown that matrix degrading invadopodia exist in tumour spheres created from primary GBM biopsy tissues [13] and Arismendi-Morillo et al. [68] utilized electron microscopy to reveal the presence of invadopodia on GBM cells in 2- to 5-mm thick tumour biopsies.

Since the majority of GBMs are known to relapse within 2 cm of the margin from the original lesion and single cell invasion can be observed in the contralateral hemisphere, this study highlights a crucial role of invadopodia and sEVs as mediators of an enhanced invasive phenotype in GBM cells that survive RT/TMZ treatment. Through proteomic evaluation of GBM cells and sEVs, we identified a variety of proteins that may contribute to the enhanced invadopodia-mediated ECM degradation in GBM cells post-RT/TMZ treatment. Importantly, we show that targeting key invadopodia-related processes, such as microtubule dynamics, can impede this enhanced invadopodia activity and sEV secretion in GBM cells that survive RT/TMZ treatment. Therefore, this may be a promising therapeutic strategy for impeding invasion of GBM cells.

## Supplementary Information

Below is the link to the electronic supplementary material.

Supplementary file1 (PDF 712 KB)

## Data Availability

Not applicable.

## Abbreviations

DAPI: 4′,6-Diamidino-2-phenylindole
DE: Differentially expressed
DMA: Dimethyl amiloride
DTT: Dithiothreitol
DNA: Deoxyribonucleic acid
ECM: Extracellular matrix
EVs: Extracellular vesicles
FBS: Fetal bovine serum
FC: Fold change
FDA: Food and Drug Administration
FDR: False discovery rate
FITC: Fluorescein isothiocyanate
h: Hour
HRP: Horseradish peroxidase
GBM: Glioblastoma multiforme
Gy: Gray
LFQ: Label free quantitation
μM: Micromolar
min: Minute
miRNA: MicroRNA
MMP: Matrix metalloproteinases
mRNA: Messenger RNA
msec: Millisecond
MT1-MMP: Matrix metalloproteinase-14
MS/MS: Tandem mass spectrometry
MTT: 3-(4,5-Dimethylthiazol-2-yl)-2,5-diphenyltetrazolium bromide
nm: Nanometre
NTA: Nanoparticle Tracking Analysis
PBS: Phosphate buffered saline
ppm: Part per million
RNA: Ribonucleic acid
RT: Radiotherapy
SD: Standard deviation
SDS: Sodium dodecyl sulfate
sEV: Small extracellular vesicle
TBST: Tris-buffered saline with 0.1% Tween 20 detergent
TCGA: The Cancer Genome Atlas
TEAB: Triethylammonium bicarbonate
TMZ: Temozolomide
TFA: Trifluoroacetic acid
VT: Vinorelbine tartrate
v/v: Volume per volume

## References

[1] Velásquez C, Mansouri S, Mora C, Nassiri F, Suppiah S, Martino J, Zadeh G, Fernández-Luna JL (2019). J. Oncol..

[2] Thakkar JP, Dolecek TA, Horbinski C, Ostrom QT, Lightner DD, Barnholtz-Sloan JS, Villano JL (2014). Cancer Epidemiol. Biomark. Prev..

[3] R. Stupp, W.P. Mason, M.J. van den Bent, M. Weller, B. Fisher, M.J.B. Taphoorn, K. Belanger, A.A. Brandes, C. Marosi, U. Bogdahn, J. Curschmann, R.C. Janzer, S.K. Ludwin , T. Gorlia, A. Allgeier, D. Lacombe, J.G. Cairncross, E. Eisenhauer and R.O. Mirimanoff, N. Engl J. Med. **352**, 987–996 (2005). 10.1056/NEJMoa043330 10.1056/NEJMoa043330 15758009

[4] D'Alterio C, Scala S, Sozzi G, Roz L, Bertolini G (2020). Semin Cancer Biol.

[5] Cordes N, Hansmeier B, Beinke C, Meineke V, van Beuningen D (2003). Br. J. Cancer.

[6] Hegedus B, Zach J, Czirok A, Lovey J, Vicsek T (2004). J. Neurooncol..

[7] Trog D, Fountoulakis M, Friedlein A, Golubnitschaja O (2006). Proteomics.

[8] Trog D, Yeghiazaryan K, Fountoulakis M, Friedlein A, Moenkemann H, Haertel N, Schueller H, Breipohl W, Schild H, Leppert D, Golubnitschaja O (2006). Eur. J. Pharmacol..

[9] Wild-Bode C, Weller M, Rimner A, Dichgans J, Wick W (2001). Implic. Radiother. Hum. Glioblastoma.

[10] Kelly T, Yan Y, Osborne R, Athota A, Rozypal T, Colclasure JC, Chu W (1998). Clin Exp Metastasis..

[11] C. Petropoulos, P.-O. Guichet, K. Masliantsev, M. Wager and L. Karayan-Tapon, Oncotarget, 20640–20657 (2018) 10.18632/oncotarget.25045 PMC5945526 29755678

[12] L. Mao, C.A. Whitehead, L. Paradiso, A.H. Kaye, A.P. Morokoff, R.B. Luwor and S.S. Stylli, J. Neurosurg. 1–13 (2017). 10.3171/2017.5.jns17845

[13] Stylli S, Kaye A, Lock P (2008). J. Clin. Neurosci..

[14] Whitehead CA, Nguyen HP, Morokoff AP, Luwor RB, Paradiso L, Kaye AH, Mantamadiotis T, Stylli SS (2018). Transl. Oncol..

[15] Xu R, Rai A, Chen M, Suwakulsiri W, Greening DW, Simpson RJ (2018). Nat. Rev. Clin. Oncol..

[16] D'Asti E, Chennakrishnaiah S, Lee TH, Rak J (2016). Cell Mol Neurobiol.

[17] Giusti I, Di Francesco M, Dolo V (2017). Curr Cancer Drug Targets.

[18] van der Vos KE, Abels ER, Zhang X, Lai C, Carrizosa E, Oakley D, Prabhakar S, Mardini O, Crommentuijn MH, Skog J (2015). Neuro Oncol.

[19] Hallal S, Mallawaaratchy DM, Wei H, Ebrahimkhani S, Stringer BW, Day BW, Boyd AW, Guillemin GJ, Buckland ME, Kaufman KL (2019). Mol Neurobiol.

[20] S.S. Stylli, S.T.T. I, A.M. Verhagen, S.S. Xu, I. Pass, S.A. Courtneidge and P. Lock, J. Cell Sci. **122**, 2727–2740 (2009). 10.1242/jcs.046680 10.1242/jcs.046680 PMC2909319 19596797

[21] A. Rai, H. Fang, M. Fatmous, B. Claridge, Q.H. Poh, R.J. Simpson and D.W. Greening, in Proteomic Profiling: Methods and Protocols, ed. by A. Posch (Springer US, New York, NY, 2021), p. 105-149

[22] K.W. Witwer, E. Aikawa, M.J. Alcaraz, J.D. Anderson, R. Andriantsitohaina, A. Antoniou, T. Arab, F. Archer, G.K. Atkin-Smith, D.C. Ayre, J.-M. Bach, D. Bachurski, H. Baharvand, L. Balaj, S. Baldacchino, N.N. Bauer, A.A. Baxter, M. Bebawy, C. Beckham, A. Bedina Zavec, A. Benmoussa, A.C. Berardi, P. Bergese, E. Bielska, C. Blenkiron, S. Bobis-Wozowicz, E. Boilard, W. Boireau, A. Bongiovanni, F.E. Borràs, S. Bosch, C.M. Boulanger, X. Breakefield, A.M. Breglio, M.Á. Brennan, D.R. Brigstock, A. Brisson, M.L.D. Broekman, J.F. Bromberg, P. Bryl-Górecka, S. Buch, A.H. Buck, D. Burger, S. Busatto, D. Buschmann, B. Bussolati, E.I. Buzás, J.B. Byrd, G. Camussi, D.R.F. Carter, S. Caruso, L.W. Chamley, Y.-T. Chang, C. Chen, S. Chen, L. Cheng, A.R. Chin, A. Clayton, S.P. Clerici, A. Cocks, E. Cocucci, R.J. Coffey, A. Cordeiro-da-Silva, Y. Couch, F.A.W. Coumans, B. Coyle, R. Crescitelli, M.F. Criado, C. D’Souza-Schorey, S. Das, A. Datta Chaudhuri, P. de Candia, E.F. De Santana, O. De Wever, H.A. del Portillo, T. Demaret, S. Deville, A. Devitt, B. Dhondt, D. Di Vizio, L.C. Dieterich, V. Dolo, A.P. Dominguez Rubio, M. Dominici, M.R. Dourado, T.A.P. Driedonks, F.V. Duarte, H.M. Duncan, R.M. Eichenberger, K. Ekström, S. El Andaloussi, C. Elie-Caille, U. Erdbrügger, J.M. Falcón-Pérez, F. Fatima, J.E. Fish, M. Flores-Bellver, A. Försönits, A. Frelet-Barrand, F. Fricke, G. Fuhrmann, S. Gabrielsson, A. Gámez-Valero, C. Gardiner, K. Gärtner, R. Gaudin, Y.S. Gho, B. Giebel, C. Gilbert, M. Gimona, I. Giusti, D.C.I. Goberdhan, A. Görgens, S.M. Gorski, D.W. Greening, J.C. Gross, A. Gualerzi, G.N. Gupta, D. Gustafson, A. Handberg, R.A. Haraszti, P. Harrison, H. Hegyesi, A. Hendrix, A.F. Hill, F.H. Hochberg, K.F. Hoffmann, B. Holder, H. Holthofer, B. Hosseinkhani, G. Hu, Y. Huang, V. Huber, S. Hunt, A.G.-E. Ibrahim, T. Ikezu, J.M. Inal, M. Isin, A. Ivanova, H.K. Jackson, S. Jacobsen, S.M. Jay, M. Jayachandran, G. Jenster, L. Jiang, S.M. Johnson, J.C. Jones, A. Jong, T. Jovanovic-Talisman, S. Jung, R. Kalluri, S.-i. Kano, S. Kaur, Y. Kawamura, E.T. Keller, D. Khamari, E. Khomyakova, A. Khvorova, P. Kierulf, K.P. Kim, T. Kislinger, M. Klingeborn, D.J. Klinke, M. Kornek, M.M. Kosanović, Á.F. Kovács, E.-M. Krämer-Albers, S. Krasemann, M. Krause, I.V. Kurochkin, G.D. Kusuma, S. Kuypers, S. Laitinen, S.M. Langevin, L.R. Languino, J. Lannigan, C. Lässer, L.C. Laurent, G. Lavieu, E. Lázaro-Ibáñez, S. Le Lay, M.-S. Lee, Y.X.F. Lee, D.S. Lemos, M. Lenassi, A. Leszczynska, I.T.S. Li, K. Liao, S.F. Libregts, E. Ligeti, R. Lim, S.K. Lim, A. Linē, K. Linnemannstöns, A. Llorente, C.A. Lombard, M.J. Lorenowicz, Á.M. Lörincz, J. Lötvall, J. Lovett, M.C. Lowry, X. Loyer, Q. Lu, B. Lukomska, T.R. Lunavat, S.L.N. Maas, H. Malhi, A. Marcilla, J. Mariani, J. Mariscal, E.S. Martens-Uzunova, L. Martin-Jaular, M.C. Martinez, V.R. Martins, M. Mathieu, S. Mathivanan, M. Maugeri, L.K. McGinnis, M.J. McVey, D.G. Meckes, K.L. Meehan, I. Mertens, V.R. Minciacchi, A. Möller, M. Møller Jørgensen, A. Morales-Kastresana, J. Morhayim, F. Mullier, M. Muraca, L. Musante, V. Mussack, D.C. Muth, K.H. Myburgh, T. Najrana, M. Nawaz, I. Nazarenko, P. Nejsum, C. Neri, T. Neri, R. Nieuwland, L. Nimrichter, J.P. Nolan, E.N.M. Nolte-’t Hoen, N. Noren Hooten, L. O’Driscoll, T. O’Grady, A. O’Loghlen, T. Ochiya, M. Olivier, A. Ortiz, L.A. Ortiz, X. Osteikoetxea, O. Østergaard, M. Ostrowski, J. Park, D.M. Pegtel, H. Peinado, F. Perut, M.W. Pfaffl, D.G. Phinney, B.C.H. Pieters, R.C. Pink, D.S. Pisetsky, E. Pogge von Strandmann, I. Polakovicova, I.K.H. Poon, B.H. Powell, I. Prada, L. Pulliam, P. Quesenberry, A. Radeghieri, R.L. Raffai, S. Raimondo, J. Rak, M.I. Ramirez, G. Raposo, M.S. Rayyan, N. Regev-Rudzki, F.L. Ricklefs, P.D. Robbins, D.D. Roberts, S.C. Rodrigues, E. Rohde, S. Rome, K.M.A. Rouschop, A. Rughetti, A.E. Russell, P. Saá, S. Sahoo, E. Salas-Huenuleo, C. Sánchez, J.A. Saugstad, M.J. Saul, R.M. Schiffelers, R. Schneider, T.H. Schøyen, A. Scott, E. Shahaj, S. Sharma, O. Shatnyeva, F. Shekari, G.V. Shelke, A.K. Shetty, K. Shiba, P.R.M. Siljander, A.M. Silva, A. Skowronek, O.L. Snyder, R.P. Soares, B.W. Sódar, C. Soekmadji, J. Sotillo, P.D. Stahl, W. Stoorvogel, S.L. Stott, E.F. Strasser, S. Swift, H. Tahara, M. Tewari, K. Timms, S. Tiwari, R. Tixeira, M. Tkach, W.S. Toh, R. Tomasini, A.C. Torrecilhas, J.P. Tosar, V. Toxavidis, L. Urbanelli, P. Vader, B.W.M. van Balkom, S.G. van der Grein, J. Van Deun, M.J.C. van Herwijnen, K. Van Keuren-Jensen, G. van Niel, M.E. van Royen, A.J. van Wijnen, M.H. Vasconcelos, I.J. Vechetti, T.D. Veit, L.J. Vella, É. Velot, F.J. Verweij, B. Vestad, J.L. Viñas, T. Visnovitz, K.V. Vukman, J. Wahlgren, D.C. Watson, M.H.M. Wauben, A. Weaver, J.P. Webber, V. Weber, A.M. Wehman, D.J. Weiss, J.A. Welsh, S. Wendt, A.M. Wheelock, Z. Wiener, L. Witte, J. Wolfram, A. Xagorari, P. Xander, J. Xu, X. Yan, M. Yáñez-Mó, H. Yin, Y. Yuana, V. Zappulli, J. Zarubova, V. Žėkas, J.-y. Zhang, Z. Zhao, L. Zheng, A.R. Zheutlin, A.M. Zickler, P. Zimmermann, A.M. Zivkovic, D. Zocco and E.K. Zuba-Surma, J. Extracell. Vesicles **7**, 1535750 (2018). 10.1080/20013078.2018.1535750

[23] Q.H. Poh, A. Rai, Carmichael, II, L.A. Salamonsen and D.W. Greening, Proteomics **21**, e2000210 (2021). 10.1002/pmic.202000210 10.1002/pmic.202000210 33860638

[24] Rai A, Greening DW, Xu R, Chen M, Suwakulsiri W, Simpson RJ (2021). Commun. Biol..

[25] B. Claridge, A. Rai, H. Fang, A. Matsumoto, J. Luo, J.R. McMullen and D.W. Greening, Proteomics **21**, e2100026 (2021). 10.1002/pmic.202100026 10.1002/pmic.202100026 33861516

[26] Kompa AR, Greening DW, Kong AM, McMillan PJ, Fang H, Saxena R, Wong RCB, Lees JG, Sivakumaran P, Newcomb AE, Tannous BA, Kos C, Mariana L, Loudovaris T, Hausenloy DJ, Lim SY (2021). Cardiovasc Res.

[27] Cox J, Neuhauser N, Michalski A, Scheltema RA, Olsen JV, Mann M (2011). J Proteome Res.

[28] Duivenvoorden HM, Rautela J, Edgington-Mitchell LE, Spurling A, Greening DW, Nowell CJ, Molloy TJ, Robbins E, Brockwell NK, Lee CS, Chen M, Holliday A, Selinger CI, Hu M, Britt KL, Stroud DA, Bogyo M, Moller A, Polyak K, Sloane BF, O'Toole SA, Parker BS (2017). J Pathol.

[29] Tyanova S, Temu T, Sinitcyn P, Carlson A, Hein MY, Geiger T, Mann M, Cox J (2016). Nat Methods.

[30] A. Rai, Q.H. Poh, M. Fatmous, H. Fang, S. Gurung, B. Vollenhoven, L.A. Salamonsen and D.W. Greening, Proteomics **21**, e2000211 (2021). 10.1002/pmic.202000211 10.1002/pmic.202000211 33634576

[31] Pathan M, Keerthikumar S, Ang CS, Gangoda L, Quek CY, Williamson NA, Mouradov D, Sieber OM, Simpson RJ, Salim A, Bacic A, Hill AF, Stroud DA, Ryan MT, Agbinya JI, Mariadason JM, Burgess AW, Mathivanan S (2015). Proteomics.

[32] O. Celiku, S. Johnson, S. Zhao, K. Camphausen and U. Shankavaram, PLoS One **9**, e101239 (2014). 10.1371/journal.pone.0101239 10.1371/journal.pone.0101239 PMC4091869 25010047

[33] Puchalski RB, Shah N, Miller J, Dalley R, Nomura SR, Yoon JG, Smith KA, Lankerovich M, Bertagnolli D, Bickley K, Boe AF, Brouner K, Butler S, Caldejon S, Chapin M, Datta S, Dee N, Desta T, Dolbeare T, Dotson N, Ebbert A, Feng D, Feng X, Fisher M, Gee G, Goldy J, Gourley L, Gregor BW, Gu G, Hejazinia N, Hohmann J, Hothi P, Howard R, Joines K, Kriedberg A, Kuan L, Lau C, Lee F, Lee H, Lemon T, Long F, Mastan N, Mott E, Murthy C, Ngo K, Olson E, Reding M, Riley Z, Rosen D, Sandman D, Shapovalova N, Slaughterbeck CR, Sodt A, Stockdale G, Szafer A, Wakeman W, Wohnoutka PE, White SJ, Marsh D, Rostomily RC, Ng L, Dang C, Jones A, Keogh B, Gittleman HR, Barnholtz-Sloan JS, Cimino PJ, Uppin MS, Keene CD, Farrokhi FR, Lathia JD, Berens ME, Iavarone A, Bernard A, Lein E, Phillips JW, Rostad SW, Cobbs C, Hawrylycz MJ, Foltz GD (2018). Science.

[34] R. Mentlein, K. Hattermann and J. Held-Feindt, Biochim. Biophys. Acta (BBA)-Rev. Cancer **1825**, 178–185 (2012) 10.1016/j.bbcan.2011.12.001 22209868

[35] V. Indira Chandran, C. Welinder, K. Gonçalves de Oliveira, M. Cerezo-Magaña, A.-S. Månsson, M.C. Johansson, G. Marko-Varga and M. Belting, J. Neuro-Oncol. **144**, 477–488 (2019) 10.1007/s11060-019-03262-4 10.1007/s11060-019-03262-4 PMC6764937 31414377

[36] Chun S, Ahn S, Yeom C-H, Park S (2016). Biology.

[37] Mallawaaratchy DM, Hallal S, Russell B, Ly L, Ebrahimkhani S, Wei H, Christopherson RI, Buckland ME, Kaufman KL (2017). J. Neurooncol..

[38] Haraszti RA, Didiot M-C, Sapp E, Leszyk J, Shaffer SA, Rockwell HE, Gao F, Narain NR, DiFiglia M, Kiebish MA (2016). J. Extracell. Vesicles.

[39] Pei J, Moon K-S, Pan S, Lee K-H, Ryu H-H, Jung T-Y, Kim I-Y, Jang W-Y, Jung C-H, Jung S (2014). Brain Tumor. Res. Treat..

[40] Pan J, Sheng S, Ye L, Xu X, Ma Y, Feng X, Qiu L, Fan Z, Wang Y, Xia X, Zheng JC (2022). Cell. Commun. Signal.

[41] E. Kiyga, Z. Adiguzel and E. Onay Ucar, Mol. Biol. Rep. **49**, 8701–8713 (2022). 10.1007/s11033-022-07714-5 10.1007/s11033-022-07714-5 35752701

[42] Chen L, Li Z, Hu S, Deng Q, Hao P, Guo S (2022). Cancer Chemother. Pharmacol..

[43] A. Rai, H. Fang, B. Claridge, R.J. Simpson, D.W. Greening, J Extracell Vesicles. **10**, e12164 (2021). 10.1002/jev2.12164 10.1002/jev2.12164 PMC8612312 34817906

[44] Beghein E, Devriese D, Van Hoey E, Gettemans J (2018). Sci. Rep..

[45] Ostermann S, Csajka C, Buclin T, Leyvraz S, Lejeune F, Decosterd LA, Stupp R (2004). Clin. Cancer Res..

[46] Mrowczynski OD, Madhankumar AB, Sundstrom JM, Zhao Y, Kawasawa YI, Slagle-Webb B, Mau C, Payne RA, Rizk EB, Zacharia BE, Connor JR (2018). Oncotarget.

[47] Arscott WT, Tandle AT, Zhao S, Shabason JE, Gordon IK, Schlaff CD, Zhang G, Tofilon PJ, Camphausen KA (2013). Transl. Oncol..

[48] Savina A, Furlán M, Vidal M, Colombo M (2003). J. Biol. Chem..

[49] Sun J, Lu F, He H, Shen J, Messina J, Mathew R, Wang D, Sarnaik AA, Chang WC, Kim M, Cheng H, Yang S (2014). J. Cell. Biol..

[50] D. Hoshino, K.C. Kirkbride, K. Costello, E.S. Clark, S. Sinha, N. Grega-Larson, M.J. Tyska and A.M. Weaver, Cell Rep. **5**, (2013). 10.1016/j.celrep.2013.10.050 10.1016/j.celrep.2013.10.050 PMC3873329 24290760

[51] Murphy D, Courtneidge S (2011). Nat. Rev. Mol. Cell Biol..

[52] Yoshioka Y, Kosaka N, Konishi Y, Ohta H, Okamoto H, Sonoda H, Nonaka R, Yamamoto H, Ishii H, Mori M, Furuta K, Nakajima T, Hayashi H, Sugisaki H, Higashimoto H, Kato T, Takeshita F, Ochiya T (2014). Nat. Commun..

[53] Zomer A, Maynard C, Verweij FJ, Kamermans A, Schafer R, Beerling E, Schiffelers RM, de Wit E, Berenguer J, Ellenbroek SIJ, Wurdinger T, Pegtel DM, van Rheenen J (2015). Cell.

[54] Hanahan D, Weinberg R (2011). Cell.

[55] Bobrie A, Thery C (2013). Biochem. Soc. Trans..

[56] Hood JL, San RS, Wickline SA (2011). Cancer Res..

[57] Martins VR, Dias MS, Hainaut P (2013). Curr. Opin. Oncol..

[58] B. Basu and M.K. Ghosh, Bioessays **41**, e1800245 (2019). 10.1002/bies.201800245 10.1002/bies.201800245 31188499

[59] J.M. Kros, D.M. Mustafa, L.J. Dekker, P.A. Sillevis Smitt, T.M. Luider and P.P. Zheng, Neuro Oncol. **17**, 343–360 (2015). 10.1093/neuonc/nou207 10.1093/neuonc/nou207 PMC4483097 25253418

[60] Steinle M, Palme D, Misovic M, Rudner J, Dittmann K, Lukowski R, Ruth P, Huber S (2011). Radiother. Oncol..

[61] Garnier D, Meehan B, Kislinger T, Daniel P, Sinha A, Abdulkarim B, Nakano I, Rak J (2018). Neuro Oncol..

[62] Keklikoglou I, Cianciaruso C, Güç E, Squadrito ML, Spring LM, Tazzyman S, Lambein L, Poissonnier A, Ferraro GB, Baer C, Cassará A, Guichard A, Iruela-Arispe ML, Lewis CE, Coussens LM, Bardia A, Jain RK, Pollard JW, De Palma M (2019). Nat. Cell Biol..

[63] Steffen A, Le Dez G, Poincloux R, Recchi C, Nassoy P, Rottner K, Galli T, Chavrier P (2008). Curr. Biol..

[64] Sakurai-Yageta M, Recchi C, Le Dez G, Sibarita J-B, Daviet L, Camonis J, D'Souza-Schorey C, Chavrier P (2008). J. Cell Biol..

[65] Ngan VK, Bellman K, Panda D, Hill BT, Jordan MA, Wilson L (2000). Cancer Res.

[66] Toso C, Lindley C (1995). Am. J. Health Syst. Pharm..

[67] Samala R, Thorsheim HR, Goda S, Taskar K, Gril B, Steeg PS, Smith QR (2016). Pharm Res.

[68] Arismendi-Morillo G (2011). Biochim. Biophys. Acta.

[69] Mitchell A, Mathew G, Jiang T, Hamdy FC, Cross SS, Eaton C, Winder SJ (2013). Prostate.

[70] Day BW, Lathia JD, Bruce ZC, D’Souza RCJ, Baumgartner U, Ensbey KS, Lim YC, Stringer BW, Akgül S, Offenhäuser C, Li Y, Jamieson PR, Smith FM, Jurd CLR, Robertson T, Inglis P-L, Lwin Z, Jeffree RL, Johns TG, Bhat KPL, Rich JN, Campbell KP, Boyd AW (2019). Acta Neuropathol..

[71] Shao G, Wang R, Sun A, Wei J, Peng K, Dai Q, Yang W, Lin Q (2018). Mol. Cancer.

[72] Zhang H, Nie W, Zhang X, Zhang G, Li Z, Wu H, Shi Q, Chen Y, Ding Z, Zhou X, Yu R (2013). PLoS One.

[73] Jiang J, Zheng M, Zhang M, Yang X, Li L, Wang S-S, Wu J-S, Yu X-H, Wu J-B, Pang X, Tang Y-J, Tang Y-L, Liang X-H (2019). Neoplasia.

[74] Sugiyama M, Hasegawa H, Ito S, Sugiyama K, Maeda M, Aoki K, Wakabayashi T, Hamaguchi M, Natsume A, Senga T (2015). Oncol Rep.

[75] Yang X, Liu Z, Li Y, Chen K, Peng H, Zhu L, Zhou H, Huang A, Tang H (2018). Int. J. Clin. Exp. Pathol..

[76] Gao W, Qiao M, Luo K (2020). Cancer Biother. Radiopharm..

[77] Tu C, Ortega-Cava CF, Winograd P, Stanton MJ, Reddi AL, Dodge I, Arya R, Dimri M, Clubb RJ, Naramura M, Wagner KU, Band V, Band H (2010). Proc. Natl. Acad. Sci. U. S. A..

[78] Chen Z, Borek D, Padrick SB, Gomez TS, Metlagel Z, Ismail AM, Umetani J, Billadeau DD, Otwinowski Z, Rosen MK (2010). Nature.

[79] Kikuchi K, Takahashi K (2008). Cancer Sci..

[80] Lindberg H, Nielsen D, Jensen BV, Eriksen J, Skovsgaard T (2004). Acta Oncol..

[81] Bradshaw AR, Wickremesekera AC, Brasch HD, Chibnall AM, Davis PF, Tan ST, Itinteang T (2016). Front. Surg..

[82] Wu Z, Wu Z, Li J, Yang X, Wang Y, Yu Y, Ye J, Xu C, Qin W, Zhang Z (2012). Tumour. Biol..

[83] Yawata T, Higashi Y, Kawanishi Y, Nakajo T, Fukui N, Fukuda H, Ueba T (2019). J. Neurooncol..

[84] Nakamura T, Katagiri T, Sato S, Kushibiki T, Hontani K, Tsuchikawa T, Hirano S, Nakamura Y (2017). Oncotarget.

[85] Brun M, Glubrecht DD, Baksh S, Godbout R (2013). J Biol Chem.

[86] Xu C, Tian G, Jiang C, Xue H, Kuerbanjiang M, Sun L, Gu L, Zhou H, Liu Y, Zhang Z, Xu Q (2019). Cell Death Dis..

[87] Bartolini A, Di Paolo D, Noghero A, Murgia D, Sementa AR, Cilli M, Pasqualini R, Arap W, Bussolino F, Ponzoni M, Pastorino F, Marchiò S (2015). Cancer Res..

[88] Huo L, Wang B, Zheng M, Zhang Y, Xu J, Yang G, Guan Q (2019). Exp. Ther. Med..

[89] O'Neill AK, Gallegos LL, Justilien V, Garcia EL, Leitges M, Fields AP, Hall RA, Newton AC (2011). J. Biol. Chem..

[90] M. Matsumoto, A. Fujikawa, R. Suzuki, H. Shimizu, K. Kuboyama, T.Y. Hiyama, R.A. Hall and M. Noda, 586, 3805–3812 (2012). 10.1016/j.febslet.2012.09.018 10.1016/j.febslet.2012.09.018 23022437

[91] Chen S, Zhang J, Chen J, Wang Y, Zhou S, Huang L, Bai Y, Peng C, Shen B, Chen H, Tian Y (2019). J. Exp. Clin. Cancer Res..

[92] X. Yuan, X. Wang, B. Gu, Y. Ma, Y. Liu, M. Sun, J. Kong, W. Sun, H. Wang, F. Zhou and S. Gao, Neoplasia (New York, N.Y.) **19**, 868–884 (2017). 10.1016/j.neo.2017.08.003 10.1016/j.neo.2017.08.003 PMC5608591 28938158

[93] Murray DW, Didier S, Chan A, Paulino V, Van Aelst L, Ruggieri R, Tran NL, Byrne AT, Symons M (2014). Br. J. Cancer.

[94] Ilboudo A, Nault J-C, Dubois-Pot-Schneider H, Corlu A, Zucman-Rossi J, Samson M, Le Seyec J (2014). BMC Cancer.

[95] Park Y, Park JM, Kim DH, Kwon J, Kim IA (2017). Oncotarget.

[96] Li Z, Hao Q, Luo J, Xiong J, Zhang S, Wang T, Bai L, Wang W, Chen M, Wang W, Gu L, Lv K, Chen J (2016). Oncogene.

[97] Zhang Z, Wang Y, Chen J, Tan Q, Xie C, Li C, Zhan W, Wang M (2016). Cancer Chemother. Pharmacol..

[98] Li H, Wang Y, Rong SK, Li L, Chen T, Fan YY, Wang YF, Yang CR, Yang C, Cho WC, Yang J (2020). Int. J. Biol. Sci..

[99] Hu Y, Ye S, Li Q, Yin T, Wu J, He J (2020). Oncol. Targets. Ther..

[100] Makowska KA, Hughes RE, White KJ, Wells CM, Peckham M (2015). Cell Rep..

[101] Hsu R-M, Tsai M-H, Hsieh Y-J, Lyu P-C, Yu J-S (2010). Mol. Biol. Cell.

[102] Zhang X, Ding Z, Mo J, Sang B, Shi Q, Hu J, Xie S, Zhan W, Lu D, Yang M, Bian W, Zhou X, Yu R (2015). Mol. Carcinog..

[103] Fan C, Tu C, Qi P, Guo C, Xiang B, Zhou M, Li X, Wu X, Li X, Li G, Xiong W, Zeng Z (2019). J. Cancer.

[104] C. Zeng, R. Yan, G. Yang, S. Xiang and F. Zhao, Biosci. Rep. 40, (2020). 10.1042/bsr20193181 10.1042/BSR20193181 PMC7295629 32478377

[105] Listik E, Toma L (2020). Oncotarget.

[106] Sumardika IW, Chen Y, Tomonobu N, Kinoshita R, Ruma IMW, Sato H, Kondo E, Inoue Y, Yamauchi A, Murata H, Yamamoto KI, Tomida S, Shien K, Yamamoto H, Soh J, Futami J, Putranto EW, Hibino T, Nishibori M, Toyooka S, Sakaguchi M (2019). Mol. Carcinog..

[107] Rodriguez-Pinto D, Sparkowski J, Keough MP, Phoenix KN, Vumbaca F, Han DK, Gundelfinger ED, Beesley P, Claffey KP (2009). Cancer Immunol. Immunother..

[108] Choi D, Montermini L, Kim D-K, Meehan B, Roth FP, Rak J (2018). Mol. Cell. Proteomics.

[109] Dornier E, Coumailleau F, Ottavi JF, Moretti J, Boucheix C, Mauduit P, Schweisguth F, Rubinstein E (2012). J. Cell Biol..

[110] Fu L, Liu N, Han Y, Xie C, Li Q, Wang E (2014). Tumour. Biol..

[111] Chen Q, Wang P, Fu Y, Liu X, Xu W, Wei J, Gao W, Jiang K, Wu J, Miao Y (2017). Oncol. Rep..

[112] Yu J, Wu S-W, Wu W-P (2017). Am. J. Transl. Res..

[113] Zheng Y, Wu C, Yang J, Zhao Y, Jia H, Xue M, Xu D, Yang F, Fu D, Wang C, Hu B, Zhang Z, Li T, Yan S, Wang X, Nelson PJ, Bruns C, Qin L, Dong Q (2020). Signal Transduct. Target. Ther..

[114] M. Sanzey, S.A. Abdul Rahim, A. Oudin, A. Dirkse, T. Kaoma, L. Vallar, C. Herold-Mende, R. Bjerkvig, A. Golebiewska and S.P. Niclou, PLoS One **10**, e0123544-e0123544 (2015). 10.1371/journal.pone.0123544 10.1371/journal.pone.0123544 PMC4416792 25932951

[115] Jian Q, Miao Y, Tang L, Huang M, Yang Y, Ba W, Liu Y, Chi S, Li C (2016). Oncotarget.

[116] Wang M, Dong Q, Wang Y (2016). Tumour. Biol..

[117] Chiang CY, Pan CC, Chang HY, Lai MD, Tzai TS, Tsai YS, Ling P, Liu HS, Lee BF, Cheng HL, Ho CL, Chen SH, Chow NH (2015). Clin. Cancer Res..

[118] Khalil AA (2007). Cancer Science.

[119] Chakraborty S, Lakshmanan M, Swa HL, Chen J, Zhang X, Ong YS, Loo LS, Akıncılar SC, Gunaratne J, Tergaonkar V, Hui KM, Hong W (2015). Nat. Commun..

[120] Hong IK, Jin YJ, Byun HJ, Jeoung DI, Kim YM, Lee H (2006). J. Biol. Chem..

[121] Zhu B, Qi L, Liu S, Liu W, Ou Z, Chen M, Liu L, Zu X, Wang J, Li Y (2017). BMC Cancer.

[122] Fukata M, Watanabe T, Noritake J, Nakagawa M, Yamaga M, Kuroda S, Matsuura Y, Iwamatsu A, Perez F, Kaibuchi K (2002). Cell.

[123] Suzuki K, Takahashi K (2008). Biochem. Biophys. Res. Commun..

[124] Li Z, Xu Y, Zhang C, Liu X, Jiang L, Chen F (2014). Int. J. Mol. Med..

[125] Goicoechea SM, Zinn A, Awadia SS, Snyder K, Garcia-Mata R (2017). J. Cell Sci..

[126] Gulvady AC, Forsythe IJ, Turner CE (2019). Mol. Biol. Cell.

[127] Yamaguchi H, Lorenz M, Kempiak S, Sarmiento C, Coniglio S, Symons M, Segall J, Eddy R, Miki H, Takenawa T, Condeelis J (2005). J. Cell Biol..

[128] Ngan E, Stoletov K, Smith HW, Common J, Muller WJ, Lewis JD, Siegel PM (2017). Nat. Commun..

[129] Nakahara H, Howard L, Thompson EW, Sato H, Seiki M, Yeh Y, Chen WT (1997). Proc. Natl. Acad. Sci. U. S. A..

[130] Linder S (2007). Trends Cell Biol..

[131] Lin CW, Sun MS, Liao MY, Chung CH, Chi YH, Chiou LT, Yu J, Lou KL, Wu HC (2014). Carcinogenesis.

[132] Li X, Liang L, Huang L, Ma X, Li D, Cai S (2015). Mol. Cancer.

[133] Lagal V, Abrivard M, Gonzalez V, Perazzi A, Popli S, Verzeroli E, Tardieux I (2014). J Cell Sci.

[134] E. Frittoli, A. Palamidessi, P. Marighetti, S. Confalonieri, F. Bianchi, C. Malinverno, G. Mazzarol, G. Viale, I. Martin-Padura, M. Garré, D. Parazzoli, V. Mattei, S. Cortellino, G. Bertalot, P.P. Di Fiore, G. Scita, A RAB5/RAB4 recycling circuitry induces a proteolytic invasive program and promotes tumor dissemination. J Cell Biol **206**, 307–328 (2014). 10.1083/jcb.201403127 10.1083/jcb.201403127 PMC4107781 25049275

[135] Wiesner C, El Azzouzi K, Linder S (2013). J. Cell Sci..

[136] Beaty BT, Condeelis J (2014). Eur. J. Cell Biol..

[137] Iizuka S, Abdullah C, Buschman MD, Diaz B, Courtneidge SA (2016). Oncotarget.

[138] Brasher MI, Martynowicz DM, Grafinger OR, Hucik A, Shanks-Skinner E, Uniacke J, Coppolino MG (2017). J. Biol. Chem..

[139] J. Ma, W. Cui, S.M. He, Y.H. Duan, L.J. Heng, L. Wang and G.D. Gao, PLoS One 7, e37297 (2012). 10.1371/journal.pone.0037297 10.1371/journal.pone.0037297 PMC3357424 22629380

[140] Y.S. Guo, R. Zhao, J. Ma, W. Cui, Z. Sun, B. Gao, S. He, Y.H. Han, J. Fan, L. Yang, J. Tang and Z.J. Luo, PLoS One **9**, e90220 (2014). 10.1371/journal.pone.0090220 10.1371/journal.pone.0090220 PMC3942417 24595049

[141] Kim YH, Kwon HJ, Kim DS (2012). J. Biol. Chem..

[142] Dekky B, Ruff M, Bonnier D, Legagneux V, Théret N (2018). Oncotarget.

[143] S. Thuault, C. Mamelonet, J. Salameh, K. Ostacolo, B. Chanez, D. Salaün, E. Baudelet, S. Audebert, L. Camoin, A. Badache, Sci Rep **10**, 6787 (2020). 10.1038/s41598-020-63926-4 10.1038/s41598-020-63926-4 PMC7176661 32321993

[144] Meng DF, Xie P, Peng LX, Sun R, Luo DH, Chen QY, Lv X, Wang L, Chen MY, Mai HQ, Guo L, Guo X, Zheng LS, Cao L, Yang JP, Wang MY, Mei Y, Qiang YY, Zhang ZM, Yun JP, Huang BJ, Qian CN (2017). J. Exp. Clin. Cancer Res..

[145] Carmona G, Perera U, Gillett C, Naba A, Law AL, Sharma VP, Wang J, Wyckoff J, Balsamo M, Mosis F, De Piano M, Monypenny J, Woodman N, McConnell RE, Mouneimne G, Van Hemelrijck M, Cao Y, Condeelis J, Hynes RO, Gertler FB, Krause M (2016). Oncogene.

[146] Zuzga DS, Pelta-Heller J, Li P, Bombonati A, Waldman SA, Pitari GM (2012). Int. J. Cancer.

[147] Abou-Kheir W, Isaac B, Yamaguchi H, Cox D (2008). J. Cell Sci..

[148] V. Marchesin, G. Montagnac and P. Chavrier, PLoS One **10**, e0121747 (2015). 10.1371/journal.pone.0121747 10.1371/journal.pone.0121747 PMC4370635 25799492

[149] Tague SE, Muralidharan V, D'Souza-Schorey C (2004). Proc. Natl. Acad. Sci. U. S. A..

[150] Markwell SM, Ammer AG, Interval ET, Allen JL, Papenberg BW, Hames RA, Castaño JE, Schafer DA, Weed SA (2019). Mol. Cancer Res..

[151] Uruno T, Liu J, Li Y, Smith N, Zhan X (2003). J. Biol. Chem..

[152] Zhang Y, Nolan M, Yamada H, Watanabe M, Nasu Y, Takei K, Takeda T (2016). Biochem. Biophys. Res. Commun..

[153] Harper K, Arsenault D, Boulay-Jean S, Lauzier A, Lucien F, Dubois CM (2010). Cancer Res..

[154] Monsky WL, Lin CY, Aoyama A, Kelly T, Akiyama SK, Mueller SC, Chen WT (1994). Can. Res..

[155] Ren XL, Qiao YD, Li JY, Li XM, Zhang D, Zhang XJ, Zhu XH, Zhou WJ, Shi J, Wang W, Liao WT, Ding YQ, Liang L (2018). Cancer Lett..

[156] Peláez R, Pariente A, Pérez-Sala Á, Larrayoz IM (2019). Cancers.

[157] R. Peláez, X. Morales, E. Salvo, S. Garasa, C. Ortiz de Solórzano, A. Martínez, I.M. Larrayoz and A. Rouzaut, PLoS One **12**, e0181579 (2017). 10.1371/journal.pone.0181579 10.1371/journal.pone.0181579 PMC5540285 28767724

[158] Yang H, Guan L, Li S, Jiang Y, Xiong N, Li L, Wu C, Zeng H, Liu Y (2016). Oncotarget.

[159] Lomakina ME, Lallemand F, Vacher S, Molinie N, Dang I, Cacheux W, Chipysheva TA, Ermilova VD, de Koning L, Dubois T, Bièche I, Alexandrova AY, Gautreau A (2016). Br. J. Cancer.

[160] Ueno H, Tomiyama A, Yamaguchi H, Uekita T, Shirakihara T, Nakashima K, Otani N, Wada K, Sakai R, Arai H, Mori K (2015). Biochem. Biophys. Res. Commun..

[161] J.D. Humphries, A. Byron, M.D. Bass, S.E. Craig, J.W. Pinney, D. Knight and M.J. Humphries, Sci Signal **2**, ra51 (2009). 10.1126/scisignal.2000396 10.1126/scisignal.2000396 PMC2857963 19738201

[162] Jerrell RJ, Parekh A (2016). Biomaterials.

