# Supplementary material for: Small extracellular vesicles promote invadopodia activity in glioblastoma cells in a therapy-dependent manner
Source: Cell Oncol (Dordr). 2023 Apr 4;46(4):909–31. doi: 10.1007/s13402-023-00786-w (PMC10356899; doi:10.1007/s13402-023-00786-w)
Supplement: Supplementary file 1 — Supplementary file1 (PDF 712 KB) [file 13402_2023_786_MOESM1_ESM.pdf]

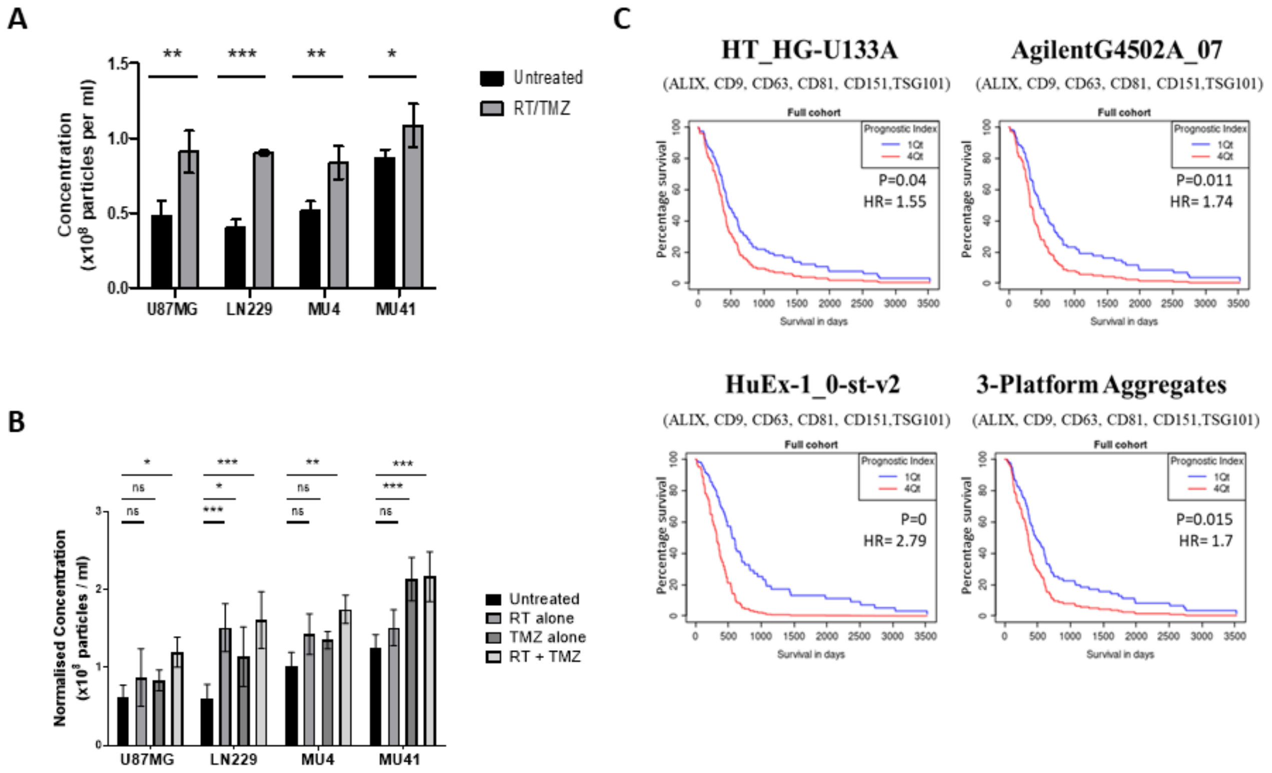

Figure S1. **RT/TMZ treatment enhances sEV secretion. (A)** The concentration of sEVs harvested from untreated and RT/TMZ treated GBM cells 24 h after treatment as determined by NTA normalized to 1 x 10^5 GBM cells (n = 3 experiments). **(B)** The concentration of sEVs harvested from untreated, RT, TMZ and RT/TMZ treated GBM cells 24 h after treatment as determined by NTA normalized to 1 x 10^5 GBM cells (n = 3 experiments). **(C)** The correlation of mRNA expression levels corresponding to exosomal markers (ALIX, CD9, CD63, CD81, CD151, TSG101) and GBM patient survival was interrogated in the Glioblastoma Bio Discovery Portal (GBM-BioDP). *P* values below *p*=0.01 are listed as ‘p-val = 0’.

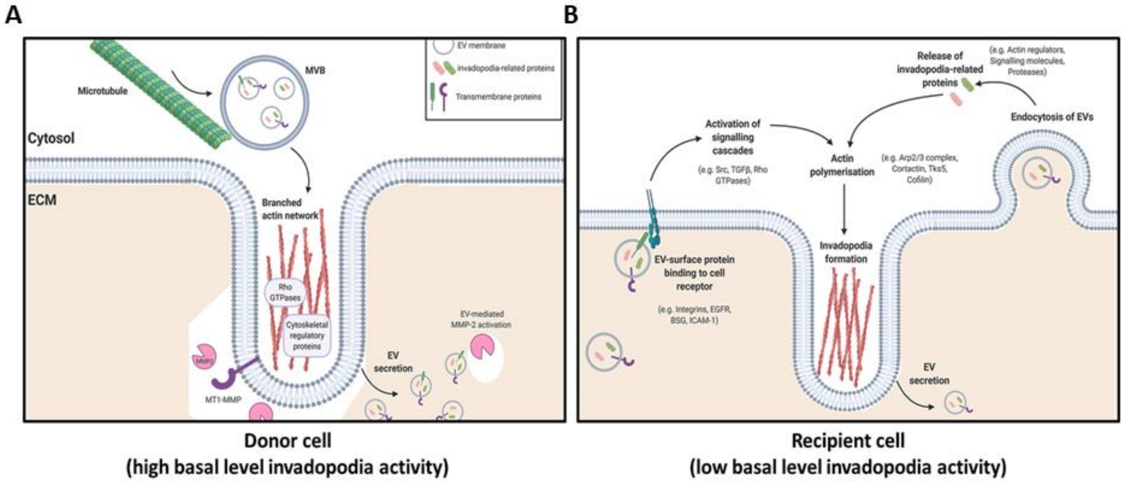

Figure S2. **Proposed interactions between sEVs and invadopodia.** A schematic representation of the sEV-mediated transfer of cargo from a high invadopodia activity GBM cell line to a recipient GBM cell line. **(A)** High invadopodia activity GBM donor cells transport key proteins required for proteolytic degradation via multi-vesicular bodies (MVBs) to mature invadopodia through interactions with the branched actin network in the invadopodium core. Once docked at the membrane the MVBs may release sEVs to promote further ECM degradation, in addition to what is achieved by invadopodia. **(B)** The sEVs were also found to be enriched in surface proteins which would allow for direct interaction with proteins on the recipient GBM cell membrane, stimulating signalling cascades to initiate invadopodia formation. In addition, the sEVs can also be endocytosed by recipient GBM cells whereby the delivery of pro-invadopodia protein cargo can lead to the promotion of invadopodia formation and activity in the recipient GBM cell.

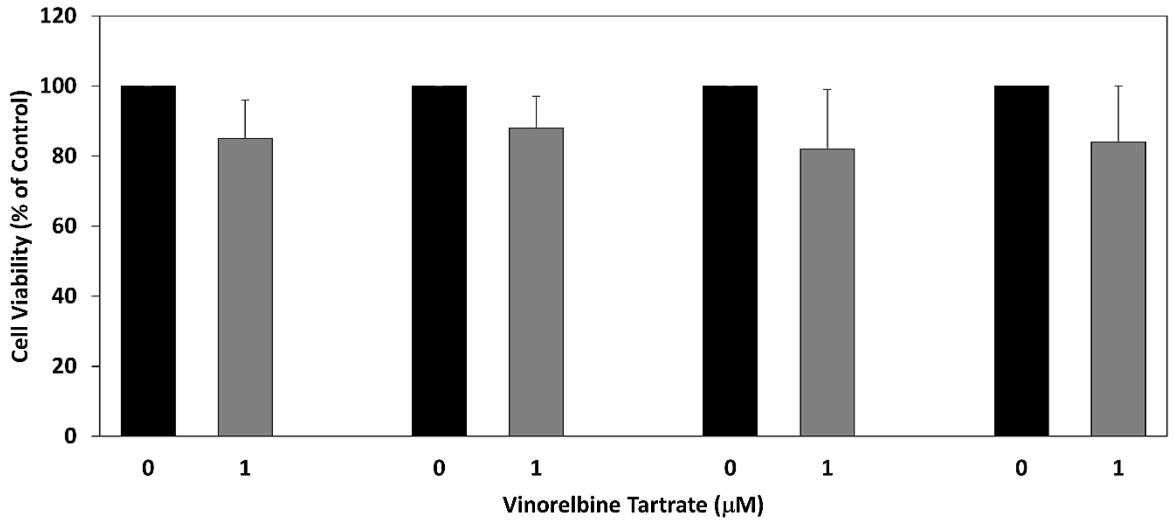

Figure S3. **Vinorelbine treatment reduces GBM cell viability.** GBM cells were treated with 1 μM VT for 24 h before a further 24 h incubation period in VT and serum free OptiMEM medium, prior to performing a cell viability test (n=3 experiments). Cell viability results of the VT treated cells are calculated relative to the untreated Control cells. GBM cell line order (left to right) : U87MG, LN229, MU4 and MU41. The reduction in GBM cell viability was not statistically significant.

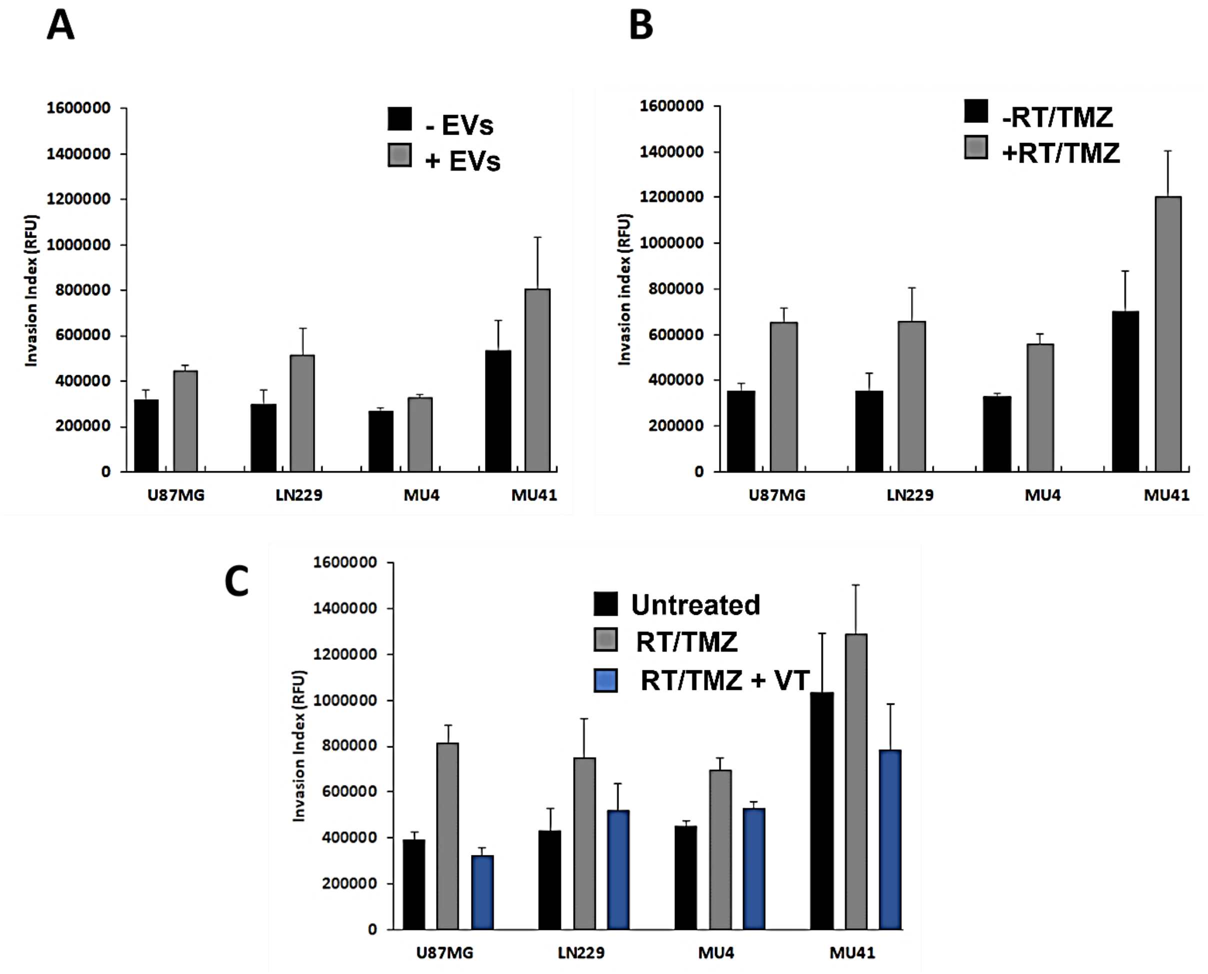

Figure S4. **3D invasion of GBM cells is enhanced with RT/TMZ or GBM cell derived sEVs, but reduced with VT treatment.**

GBM cells were incubated with LN229 sEVs or treated with RT/TMZ 24 h prior to being seeded into a commercial Cultrex BME Cell Invasion assay and processed as per the manufacturer's instructions following a 24 h incubation. It can be observed that the pre-incubation with LN229 GBM cell sEVs **(A)** or **(B)** RT//TMZ treatment enhances the 3D invasive capacity of the GBM cells. However, VT treatment (1 μM) applied at the same time as RT/TMZ treatment reduced the enhanced invasion observed with just RT/TMZ treatment alone. Each group was set up in triplicate wells and each experiment was also performed in triplicate. (Results are presented as mean ± SD; pairwise comparisons between RT/TMZ and RT/TMZ + VT are $p < 0.05$; unpaired student's t-test).

A

3-Platform Aggregates	*Full Cohort*	*C subtype*	*M subtype*	*P subtype*	*N subtype*
p-value	0	0	0	0.001	-
Hazard Ratio	3.03	3.22	16.95	9.15	MDC*
**HT_HG-U133A**	*Full Cohort*	*C subtype*	*M subtype*	*P subtype*	*N subtype*
p-value	0	0	0	0.001	-
Hazard Ratio	3.28	13.17	16.95	9.15	MDC*
**AgilentG4502A_07**	*Full Cohort*	*C subtype*	*M subtype*	*P subtype*	*N subtype*
p-value	0	0	0.001	0	-
Hazard Ratio	2.51	16.55	6.74	11.77	MDC*
**HuEx-1_0-st-v2**	*Full Cohort*	*C subtype*	*M subtype*	*P subtype*	*N subtype*
p-value	0	0	0.001	0.001	-
Hazard Ratio	2.31	13.44	10.22	34.19	MDC*
Hazard Ratio Average		11.595	12.715	16.065	

B

3-Platform Aggregates	*Full Cohort*	*C subtype*	*M subtype*	*P subtype*	*N subtype*
p-value	0	0.001	0	0.002	-
Hazard Ratio	3.03	6.33	15.49	5.42	MDC*
**HT_HG-U133A**	*Full Cohort*	*C subtype*	*M subtype*	*P subtype*	*N subtype*
p-value	0	0.007	0	0.001	0.037
Hazard Ratio	3.28	4.09	12.76	15.88	11.51
**AgilentG4502A_07**	*Full Cohort*	*C subtype*	*M subtype*	*P subtype*	*N subtype*
p-value	0	0.001	0.002	0.001	-
Hazard Ratio	2.51	14.18	4.52	6.62	MDC*
**HuEx-1_0-st-v2**	*Full Cohort*	*C subtype*	*M subtype*	*P subtype*	*N subtype*
p-value	0	0.001	0	0	0.031
Hazard Ratio	2.31	9.5	12.02	21.3	11.02
Hazard Ratio Average		8.525	11.1975	12.305	11.265

C

3-Platform Aggregates	*Full Cohort*	*C subtype*	*M subtype*	*P subtype*	*N subtype*
p-value	0	0	0	0	-
Hazard Ratio	3.03	11.64	12.96	11.66	MDC*
**HT_HG-U133A**	*Full Cohort*	*C subtype*	*M subtype*	*P subtype*	*N subtype*
p-value	0	0	0	0.001	-
Hazard Ratio	3.28	10.42	7.04	20.68	MDC*
**AgilentG4502A_07**	*Full Cohort*	*C subtype*	*M subtype*	*P subtype*	*N subtype*
p-value	0	0	0	0.001	-
Hazard Ratio	2.51	13.39	11.37	39.6	MDC*
**HuEx-1_0-st-v2**	*Full Cohort*	*C subtype*	*M subtype*	*P subtype*	*N subtype*
p-value	0	0.003	0	0.001	-
Hazard Ratio	2.31	5.16	18.72	32.53	MDC*
Hazard Ratio Average		10.1525	12.5225	26.1175	

D

3-Platform Aggregates	*Full Cohort*	*C subtype*	*M subtype*	*P subtype*	*N subtype*
p-value	0	0	0.001	0	0
Hazard Ratio	3.03	3.75	2.79	3.5	8.6
**HT_HG-U133A**	*Full Cohort*	*C subtype*	*M subtype*	*P subtype*	*N subtype*
p-value	0	0.001	0.002	0	0.002
Hazard Ratio	3.28	3.05	2.54	4.17	4.49
**AgilentG4502A_07**	*Full Cohort*	*C subtype*	*M subtype*	*P subtype*	*N subtype*
p-value	0	0	0.001	0.001	-
Hazard Ratio	2.51	6.59	7.46	15.17	MDC*
**HuEx-1_0-st-v2**	*Full Cohort*	*C subtype*	*M subtype*	*P subtype*	*N subtype*
p-value	0	0.001	0.001	0.001	-
Hazard Ratio	2.31	6.59	7.46	15.17	mdc*
Hazard Ratio Average		4.995	5.0625	9.5025	6.545

Figure S5: **Invasion and invadopodia related proteins isolated from GBM cells /EVs post-treatment impacts GBM patient survival.** Invasion and invadopodia genes that were analyzed using the 'Full Cohort' of GBM-BioDip for their impact on GBM patient survival were also assessed using the four GBM subtypes – Classical (C subtype), Mesenchymal (M subtype), Proneural (P subtype) and Neural (N subtype) across the datasets (Affymetrix HGU133A, Agilent G4502A, HuEx-1_0-st-v2, 3-Platform Aggregates). A Cox proportional hazards model was constructed based on a prognostic index generated from the combined expression levels of the interrogated genes and data was stratified according to the lowest quartile versus the highest quartile. *P* values below *p* = 0.01 are listed as 'p-val=0'. The Cox model was not always able to be computed and was considered non-convergent (MDC). Analyses are presented for (A) invasion and (B) invadopodia genes for the corresponding proteins which were increased in GBM cells following RT/TMZ treatment and (C) invasion and (D) invadopodia genes in sEVs.

Supplementary Table 1

Table S1: Invasion-related targets of miRNAs with reduced expression in recipient MU4 GBM cells following incubation with LN229 GBM cell line derived sEVs

miRNA species	Strongly Validated	Weakly Validated	Predicted >95 target score	Fold change
**hsa-miR-4454+ hsa-miR-7975**	–	–	–	-5.741
**hsa-miR-365a-3p+hsa-miR-365b-3p**	IL6, **KRAS**, RHOH	**AKT1**, THBS2	–	-4.107
**hsa-miR-149-5p**	IL6	ADAM17	–	-3.679
**hsa-miR-129-5p**	–	ADAMTS4, ADAM17, ADAM12, FIGN	–	-3.585
**hsa-miR-27b-3p**	**EGFR**, MET, MMP13, THBS1, THBS2	ADAMTS5**, WASL**	ADAMTS10	-3.555
**hsa-miR-181a-5p**	KRAS, PTEN, NRAS, STAT3	ADAM17, **RHOG**	ADAM11, FIGN, IL2,	-3.422
**hsa-miR-24-3p**	IL4, IL18, **MMP14**, MYC, **SH3PXD2A**, TP53	ADAM17	–	-3.330
**hsa-miR-155-5p**	MMP16, MYC, **RHOA**, **SH3PXD2A**, STAT1	ADAM10, ADAMTS4, STAT3	–	-3.294
**hsa-miR-129-2-3p**	MYC	THBS2	–	-3.183
**hsa-miR-34a-5p**	**CD44**, IL10, MET, MMP7, MYC, TP53, VEGFA	**CTTN,** STAT1	–	-3.137
**hsa-miR-204-5p**	IL11, JAK2, **MMP9**	MMP3	–	-3.011
**hsa-miR-93-5p**	PTEN, MMP3, MYC, **RHOC**	**GRB2, SH3PXD2A**, TSG101, STAT3, **WASL**	–	-2.946
**hsa-miR-221-3p**	ADAM1A**, MMP2, PAK1, PTEN**, STAT5A	**RHOA**		-2.800
**hsa-miR-222-3p**	ADAM1A, **GRB10**, MMP1, **PTEN**, P53, STAT5A,	–	–	-2.774
**hsa-miR-9-5p**	IL6, MMP13, **MMP2**, **MMP9**	–	–	-2.657
**hsa-let-7d-5p**	IL13	FIGN, THBS1, **WASL**	ADAMTS8, FIGNL2	-2.594
**hsa-miR-25-3p**	MYC, **PTEN**, TP53	**WASL**	ADAM19	-2.510
**hsa-miR-22-3p**	**BSG**, **CD151**, **PTEN**	–	–	-2.471

**hsa-let-7a-5p**	**EGFR**, IL2, **KRAS**, THBS1	COL8A1, FIGN, **WASL**	ADAMTS8, COL3A1, COL1A2, FIGN, TGFBR1	-2.375
**hsa-miR-125b-5p**	**CD44, EGFR,** MMP-13**, MMP-2,** MMP-26**,** TP53	ADAMTS1	RAB3D	-2.315
**hsa-miR-23a-3p**	SMAD3, STAT3	–	MET, STAT5B, TGFBR2	-2.279
**hsa-miR-99b-5p**	–	–	–	-2.232
**hsa-miR-199a-3p+hsa-miR-199b-3p**	MET	–	ADAM10, ADAMTS3, SERPINE2	-2.224
**hsa-miR-125a-5p**	ADAM9, **AKT1**, DOCK3, **EGFR**, JAK2, MMP11, MTOR, SMAD2, SMAD4, STAT3, VEGFA	–	–	-2.196
**hsa-let-7e-5p**	**MMP9**	FIGN, STAT3, THBS1, **WASL**	FIGNL2, NRAS	-2.099
**hsa-miR-98-5p**	IL10, IL6, MYC, NRAS, THBS1	FIGN, **WASL**	ADAMTS8	-2.027
**hsa-let-7i-5p**	IL13, IL2	FIGN, TGFBR3, THBS1, STAT2, **WASL**	ADAMTS8, FIGNL2, TGFBR1	-1.984
**hsa-let-7b-5p**	–	**KRAS**, RAB38**, RHOB, RHOG,** THBS1	ADAMTS8, COL1A2, COL3A1, FIGN, TGFBR1/3	-1.961
**hsa-miR-130a-3p**	MET, MYC, **PTEN**	**GRB10, WASL**	–	-1.882
**hsa-miR-29a-3p**	ADAM12, ADAMTS9, **CDC42, ITGA6, ITGB1, MMP2**, MYC, **PTEN,** VEGFA	TGFB3	ADAMTS17, ADAMTS7, **SH3PXD2A**	-1.856
**hsa-miR-23b-3p**	MET, **PTEN**, **SRC**, VCAN	ADAM17, ADAM28, DOCK4, VCAM1	ADAM23, STAT5B, TGFBR2	-1.836
**hsa-miR-191-5p**	–	–	–	-1.811
**hsa-let-7c-5p**	IL6, IL10, MTOR, MYC, STAT3, TGFBR1	STAT2, VCAM1, **WASL**	–	-1.655
**hsa-miR-15b-5p**	**MMP9**	**BSG**	–	-1.650

Experimentally validated (as listed in the MirTarBase database) and predicted (as listed in the miRDB database) invasion-related targets of the 34 downregulated miRNAs in MU4 cells incubated with LN229 cell line sEVs. Targets linked to invadopodia activity are highlighted in bold. Strongly validated methods: reporter assays, western blot and qPCR. Weakly validated methods: microarray, NGS, and pSILAC. All predicted targets are listed above a target score of 95, indicating that predicted targets are highly likely to bind to the listed miRNA. The miRNAs are listed in order of greatest fold change.

Supplementary Table 2

Table S2: Common invasion-related proteins that are increased in GBM cell lines and sEVs following exposure to RT/TMZ treatment

GBM Cells		
**Protein**	**Known role/s**	**Reported role in GBM**
**JUP**	Junction plakoglobin was found to be overexpressed in OSC and promoted the proliferation, migration, and invasion of OSCC cells but inhibited apoptosis	NA
**DAG1**	High expression of dystroglycan promoted prostate cancer cell invasion, but inhibited growth on soft agar [69]	Dystroglycan-receptor interactions promote a mesenchymal-like state and maintain GSCs in perivascular niches via anchoring to laminin and regulation of ERK signalling [70]
**NEDD4**	NEDD4 is significantly correlated with tumour metastasis and required for migration and invasion signalling of EGFR in gastric cancer cells [71]	NEDD4 promotes the invasion of malignant glioma cells *in vitro* via triggering ubiquitination of cyclic nucleotide Ras guanine nucleotide exchange factor (CNrasGEF) [72]
**PRRX1**	PRRX1-induces EMT (invasion/migration) but reduces cell proliferation and apoptosis of HNSCC [73]	Activation of Notch signalling by PRRX1 promotes GBM cell invasion and neurosphere formation *in vitro* [74]
**RAB5A**	Rab5a promotes the migration and invasion of hepatocellular carcinoma by up-regulating Cdc42 [75]	Overexpression of Rab5a was found to promote radio resistance and autophagy in glioma cells *in vitro* [76]
**VPS28**	VPS28 is a subunit of the ESCRT-I complex, required for the translocation of active Src from late endosomal/lysosomal compartment to focal adhesions, where Src functions to promote cell motility [77]	NA
**ABI2**	ABI2 is a regulator of actin cytoskeleton dynamics underlying cell motility and adhesion. It functions as a component of the WAVE2 complex, which activates actin nucleating machinery Arp2/3 to drive invadopodia formation [78, 79]	NA
**ACE**	ACE-inhibitors inhibit proliferation and invasion of cancer cells *in vitro* and tumour growth and metastasis *in vivo,* via the inhibition of MMP activity and VEGF expression [80]	ACE expression was detected via IHC staining on the endothelium of microvessels within GBM tissue samples [81]
**MCAM**	Silencing MCAM in SKOV-3 ovarian cancer cells reduced invasion, which correlated with decreased Rho GTPase (Cdc42 and RhoA) activation [82]	MCAM is upregulated in WHO grade III and IV gliomas, as determined by IHC staining. MCAM is also highly expressed in GSCs, and ectopic expression in differentiated GBM cells results in cell

		cycle arrest [83]
**PPP3CA**	PPP3CA is bound by C16orf74 to promote invasion in aggressive pancreatic cancers [84]	PPP3CA encodes calmodulin-binding catalytic subunit of calcineurin. Calcineurin expression was found to be elevated in areas of high infiltration/migration in GBM tissue [85]
**NPTXR**	NPTXR is a receptor for NPTX, which has been demonstrated to promote CRC metastasis through the activation of the Wnt/β-catenin signalling pathway [86]. It has also been correlated with Neuroblastoma tumour burden [87]	Ligands of NPTXR, NPTX1/2, are reported to cause dysfunction PI3K/AKT/mTOR signalling thereby affecting tumour progression in the U251 human glioma cell line [88]
**sEVs**		
**Protein**	**Known role/s**	**Reported role in GBM**
**DLG1**	Interaction of Protein Kinase C-α with DLG1 promotes cellular invasion in non-small cell lung cancer lines [89]	DLG1, also known as synapse-associated protein 97 (SAP97), was found to contribute to the stability of sodium (Na_x) channels in the plasma membrane of C6 GBM cells [90]
**RER1**	RER1 enhances the progression of prostate cancer through promoting cell proliferation, migration and invasion [91]	NA
**DOCK7**	Inhibition of DOCK7 in ESCC cells expressing the oncoprotein SET reduced invasion through a 3D matrix and directional migration *in vitro* [92]	Acts as a GEF for Rac1 to promote HGF-dependent GBM cell invasion. Furthermore, depletion of Dock7 significantly decreased HGF-induced lamellipodia formation [93]
**PI4KA**	HCC patients who had been treated by surgical resection and had higher PI4KA mRNA concentrations in their tumour tissue exhibited a higher risk of tumour recurrence (median time: 20 months versus 49 months, P = 0.0012) [94]	Inhibition of PI4K IIIα using RNAi inhibited U251 cell migration and invasion *in vitro*, whilst its inhibition in U251 xenograft mice, using an agent known as simeprevir, also had a radiosensitizing effect [95]
**HDAC2**	Elevated expression of HDAC2 can promote the migration and invasion of non-small cell lung cancer cells via up regulation fibronectin through the activation of NF-κB signalling [96].	siRNA silencing of HDAC2 suppresses the *in vitro* proliferation, migration, and invasion of GBM U87MG and A172 cell lines, and increases the sensitivity of GBM cells to TMZ [97]
**ITGA1**	ITGA1 can promote CRC cell migration, invasion and tumorigenicity by activating the Ras/Erk signalling [98]	Overexpression of the long noncoding RNA 'HULC' increased GBM cell proliferation, invasion, and migration, which correlated with an upregulation of ITGA1 protein expression [99]
**MYO18A**	MYO18A is overexpressed in highly metastatic prostate cancer cell lines, and knockdown reduced	MYO18A associates with GOLPH3 to promote Golgi extension and vesicle

	the number of filopodia formed by these cells [100]. MYO18A colocalises with PAK2 in lamellipodia to promote epithelial cell migration [101]	release, resulting in the secretion of growth factors and MMPs [4]. GOLPH3 promotes GBM cell migration and invasion via the mTOR signalling pathway [102]
**GPC6**	GPC6 promotes the migration, invasion, and proliferation of nasopharyngeal carcinoma cells in vitro [103]. Hedgehog signalling activation is also required for GPC6-mediated regulation of invasion, migration, and EMT in gastric cancer cells [104]	GPC6 was found to be expressed in the U251MG GBM cell line [105]
**NPTN**	NPTNβ can induce anchorage-independent growth, motility and invasiveness in lung cancer cells in response to extracellular S100A8/A9 [106]. Significantly higher NPTN immunoreactivity was detected in invasive breast tumour tissue compared to control breast tissue, suggesting that NPTN expression may promote tumour invasion [107]	NPTN is upregulated in EVs released from U373 GBM cells expressing EGFRvIII [108]
**TSPAN14**	Regulates ADAM10 trafficking and activity [109], which in turn can promote cell proliferation, migration, and invasion [110]	NA
**TPD52L2**	Knockdown of TPD52L2 suppressed PIK3CA/AKT signalling and suppressed the proliferation, migration and invasion of human pancreatic adenocarcinoma cells [111]	miR-485-5p downregulation of TPD52L2 reduced GBM cell proliferation, migration, and invasion *in vitro* [112]
**ENO2**	ENO2 is overexpressed in pancreatic ductal adenocarcinoma (PDAC) tissue and correlates with metastasis and poor prognosis in PDAC patients [113]	ENO2 is upregulated in hypoxic GBM cells and knockdown of ENO2 *in vivo* results in a survival benefit in mice [114]
**RAB23**	Rab23 promotes squamous cell carcinoma cells migration and invasion by regulating the Integrin β1/Rac1 pathway [115]	Rab23 is highly expressed in grade III and IV astrocytomas and promotes invasion via Rac1 activation in GBM cells [116]
**SH3BGRL3**	SH3BGRL3 promotes EMT, migration and proliferation of urothelial carcinoma *in vitro* via interaction with pEGFR through Grb2, which activates the Akt- signalling pathway [117]	Proteomic analysis identified that SH3BGRL3 is upregulated in GBM tissue compared to non-tumour brain tissue [118]

NA: not applicable

Supplementary Table 3

Table S3: DE invadopodia-related proteins in GBM cells and sEVs post-RT/TMZ treatment

GBM Cells					
**Gene**	**Protein**	**Invadopodia related evidence**	**Fold change (treated/untreated cells)**		
			**LN229**	**MU4**	**MU41**
**ABI2**	Abl interactor 2	Regulator of actin cytoskeleton dynamics underlying cell motility and adhesion. Functions as a component of the WAVE2 complex, which activates actin nucleating machinery Arp2/3 to drive invadopodia formation. [78, 79]	24.44	NS	25.51
**AGRN**	Agrin	Secreted and cell surface Agrin binds the Lrp4 receptor and promotes the formation of a Agrin–Lrp4–MuSK signalling complex, which activates FAK and Arp2/3 associated invadopodia formation [119]	22.76	NS	NS
**CD151**	CD151 antigen	CD151, together with integrin α3β1 located at invadopodia, was found to regulate expression and activity of MMPs [120]	25.15	NS	NS
**CLASP2**	CLIP-associating protein 2	Microtubule stabilising protein involved in the regulation of cell protrusions such as podosomes and invadopodia [121]	24.09	NS	25.29
**CLIP1**	CAP-Gly domain-containing linker protein 2	Also known as CLIP-170. Microtubule binding protein that forms a complex with RAC1/Cdc42/IQGAP1 and is involved in the regulation of invadopodia formation and invasion in human breast cancer cells [122, 123]	26.37	NS	NS
**DIAPH1**	Protein diaphanous homolog 1	Actin nucleation and elongation factor required for the assembly of F-actin structures. DIAPH1 was found to be critical for invadopodia formation in U87MG cells [124]	NS	24.14	NS
**DOCK1**	Dedicator of cytokinesis protein 1	Rac1 exchange factor that is localised to invadopodia, involved in modulating invadopodia dynamics in breast cancer cells [125]	NS	NS	25.63
**FHOD1**	FH1/FH2 domain-containing protein 1	Superresolution microscopy revealed the presence of formin FHOD1 and unbranched radial F-actin fibers emanating from invadopodia [126]	24.45	NS	NS
**GRB2**	Growth factor receptor-bound protein 2	Upstream activator of N-WASP in invadopodia formation [127]	NS	NS	23.18
**LPP**	Lipoma-preferred partner	Src-mediated LPP phosphorylation at specific tyrosine residues (Y245/301/302) is critical for invadopodia formation in breast cancer cells [128]	24.76	NS	NS
**MMP14**	Matrix metalloprot	MT1-MMP: transmembrane protease located within invadopodia [129]	NS	-26.17	NS

	einase-14				
**MMP2**	72 kDa type IV collagenase	Metalloprotease that is widely associated with invadopodia [130]	NS	-23.69	NS
**PODXL**	Podocalyxin	Podocalyxin-like 1 promotes invadopodia formation and metastasis through activation of Rac1/Cdc42/cortactin signalling in breast cancer cells [131]	26.38	NS	NS
**PPP4C**	Serine/threonine-protein phosphatase 4 catalytic subunit	Protein phosphatase that upregulates MMP-2 and MMP-9 via pAKT [132]	24.94	NS	NS
**RAB3A**	Ras-related protein Rab-3A	Rab3A GTPase is a key player in regulating exocytosis of vesicles containing MMPs at invadopodia [133]	NS	23.88	NS
**RAB5A**	Ras-related protein Rab-5A	Rab5a GTPase is a major regulator of MT1-MMP trafficking and invadopodia formation [134]. MT1-MMP vesicles that are positive for Rab5a localise to invadopodia [135]	25.09	NS	25.44
**RHOA**	Transforming protein RhoA	RhoA–ROCK signalling promotes invadopodia formation and activity [136]	-2.68	NS	2.66
**SH3PXD2B**	SH3 and PX domain-containing protein 2B	Adaptor protein also known as Tks4 that is required for functional invadopodia formation [137]	23.77	NS	NS
**STX4**	Syntaxin-4	SNARE protein syntaxin4: mediates trafficking of MT1-MMP and EGFR for invadopodia activity [138]	24.23	NS	NS
**TGFBI**	Transforming growth factor-beta-induced protein ig-h3	A transforming growth factor beta (TGFβ) inducible secreted extracellular matrix (ECM) protein. It has been shown to promote MMP secretion at invadopodia by interacting with integrin α2β1 and activating PI3K/AKT signalling. Processing of TGFBI by MMP-2 induces adhesion-related FAK/Src signalling in glioma cells and promotes invasion [139-141]	NS	NS	25.74
**TJP1**	Tight junction protein ZO-1	Tight Junction Protein is a scaffolding protein that plays a role in the regulation of cell migration by targeting CDC42BPB to the leading edge of migrating cells. Plays a role as a partner for ADAM-12 in invadopodia [142, 143]	24.3	NS	2.03
**TRIP10**	Cdc42-interacting protein 4	CDC42-interacting protein 4: promotes metastasis of nasopharyngeal carcinoma by mediating invadopodia formation and activating EGFR signalling. Also promotes CDC42-induced actin polymerization by recruiting WASL/N-WASP which in turn activates the Arp2/3 complex [144]	NS	NS	-24.64
**VASP**	Vasodilator-stimulated phosphoprotein	Actin-associated protein that stimulates actin filament elongation by promoting the transfer of profilin-bound actin monomers onto the barbed end of growing actin filaments. In colon cancer cells, VASP is found within	NS	NS	-25.41

		invadopodia, where it colocalizes with cortactin and actin [145, 146]			
**WASF2**	Wiskott-Aldrich syndrome protein family member 2	Promotes formation of actin filaments. Part of the WAVE complex that activates actin nucleating machinery Arp2/3 to drive invadopodia formation [79, 147]	NS	NS	-25.25
**sEVs**					
**Gene**	**Protein**	**Invadopodia related evidence**	**Fold change (treated/untreated cells)**		
			**LN229**	**MU4**	**MU41**
**AGRN**	Agrin	Secreted and cell surface Agrin binds the Lrp4 receptor and promotes the formation of a Agrin–Lrp4–MuSK signalling complex, which activates FAK and Arp2/3 associated invadopodia formation [119]	NS	NS	-25.16
**ARF6**	ADP-ribosylation factor 6	ARF6 has an established role in invadopodia. It promotes the formation of Rac1 and WAVE dependent ventral F-actin rosettes in breast cancer cells in response to epidermal growth factor. ARF6 also localizes to invadopodia in melanoma cells, where it regulates invadopodia activity through the activation of the MEK/ERK signalling pathway [148, 149]	NS	NS	2.45
**CD151**	CD151 antigen	CD151, together with integrin α3β1 located at invadopodia, was found to regulate expression and activity of MMPs [120]	NS	24.88	NS
**CLIP1**	CAP-Gly domain-containing linker protein 2	Also known as CLIP-170. Microtubule binding protein that forms a complex with RAC1/Cdc42/IQGAP1 and is involved in the regulation of invadopodia formation and invasion in human breast cancer cells [122, 123]	-25.43	NS	NS
**CSNK2A1**	Casein kinase II subunit alpha	Subunit of Casein Kinase 2. Casein Kinase 2 phosphorylates cortactin and regulates Arp2/3 activity and subsequent invadopodia function [150]	-2.50	NS	-2.36
**CSNK2A2**	Casein kinase II subunit alpha	Subunit of Casein Kinase 2. Casein Kinase 2 phosphorylates cortactin and regulates Arp2/3 activity and subsequent invadopodia function [150]	NS	24.67	25.44
**CTTN**	Src substrate cortactin	Cortactin is a critical regulator of actin nucleation in the development of invadopodia [151]	-24.78	NS	25.54
**DNM2**	Dynamin-2	Dynamin2 GTPase is a microtubule-associated force-producing protein that contributes to invadopodia formation in invasive bladder cancer cells via its proline/arginine-rich domain (PRD) [152]	-27.25	25.12	NS
**DOCK1**	Dedicator of cytokinesis protein 1	Rac1 exchange factor that is localised to invadopodia, involved in modulating invadopodia dynamics in breast cancer cells [125].	NS	25.83	NS

**ENPP2**	Ectonucleotide pyrophosphatase/phosphodiesterase family member 2	Can induce invadopodia formation in the fibrosarcoma cell line HT1080 through activation of the LPA4 receptor [153]	2.21	25.46	NS
**FAP**	Prolyl endopeptidase FAP	Also known as Seprase, a gelatin-degrading membrane-protease complex that is expressed at the invasive front of malignant melanoma cells on invadopodia [154]	NS	NS	2.19
**FMNL2**	Formin-like protein 2	Cortactin directly binds to FMNL2 to promote actin polymerization and recycling endosome motility. FMNL2 was necessary for invadopodia formation and function in CRC cells [155]	NS	NS	25.76
**FSCN1**	Fascin	Actin bundling protein that stabilises actin filaments in invadopodia [44]	NS	23.80	NS
**ICAM1**	Intercellular adhesion molecule 1	A cell adhesion molecule in the ICAM superfamily, known for their ability to bind integrins to activate signalling. Often expressed at the cell surface where it may play a role in invadopodia maturation via interactions with integrins [156]	2.22	NS	NS
**ITGA3**	Integrin alpha-3	Integrin alpha-3/beta-3 provides a docking site for FAP (seprase) at invadopodia plasma membranes in a collagen-dependent manner and hence may participate in the adhesion, formation of invadopodia and matrix degradation processes [157]	NS	-2.70	NS
**LPP**	Lipoma-preferred partner	Src-mediated LPP phosphorylation at specific tyrosine residues (Y245/301/302) is critical for invadopodia formation in breast cancer cells [128]	NS	24.63	NS
**MMP14**	Matrix metalloproteinase-14	MT1-MMP: transmembrane protease located within invadopodia [129]	-25.74	25.6	NS
**MTOR**	Serine/threonine-protein kinase mTOR	Cav-1 activation-induces PI3K/Akt/mTOR signalling to promote invadopodia formation in breast carcinoma MDA-MB-231 cells [158]	NS	NS	24.16
**NCKAP1**	Nck-associated protein 1	A subunit of the Arp2/3-WAVE complex involved in actin organisation during invadopodia formation [159]	25.38	NS	NS
**PXN**	Paxillin	A cytoskeletal protein involved in actin-membrane attachment at sites of cell adhesion to the ECM. JNK-paxillin signalling can promote invadopodia activity [160]	-24.49	24.43	25.01
**RAB5A**	Ras-related protein Rab-5A	Rab5a GTPase is a major regulator of MT1-MMP trafficking and invadopodia formation [134]. MT1-MMP vesicles that are positive for Rab5a localise to invadopodia [135]	NS	NS	-25.46
**RCC2**	Protein RCC2	Acts as a regulator of Rac1 and Arf6, as well as binding to cortactin, linking RCC2 to number of key invadopodia proteins [161]	-26.18	25.06	NS
**ROCK2**	Rho-	Protein kinase which is a key regulator of	NS	25.38	NS

	associated protein kinase 2	actin cytoskeleton. Matrix rigidity can drive ROCK signalling to enhance invadopodia function [162]			
**TGFBI**	Transforming growth factor-beta-induced protein ig-h3	A transforming growth factor beta (TGFβ) inducible secreted extracellular matrix (ECM) protein. It has been shown to promote MMP secretion at invadopodia by interacting with integrin α2β1 and activating PI3K/AKT signalling. Processing of TGFBI by MMP-2 induces adhesion-related FAK/Src signalling in glioma cells and promotes invasion [139-141]	NS	25.52	NS
**TJP1**	Tight junction protein ZO-1	Tight Junction Protein is a scaffolding protein that plays a role in the regulation of cell migration by targeting CDC42BPB to the leading edge of migrating cells. Plays a role as a partner for ADAM-12 in invadopodia [142, 143]	-24.89	NS	NS
**WASF2**	Wiskott-Aldrich syndrome protein family member 2	Promotes formation of actin filaments. Part of the WAVE complex that activates actin nucleating machinery Arp2/3 to drive invadopodia formation [79, 147]	NS	NS	24.81

Significantly changed DE proteins above a threshold of FC ±≥2 (averaged log2-transformed protein intensity identified in RT/TMZ treated cells relative to the corresponding untreated cell line) (*NS: not significant)*
